# Inhibition of Cysteine Proteases by 6,6′-Dihydroxythiobinupharidine (DTBN) from *Nuphar lutea*

**DOI:** 10.3390/molecules26164743

**Published:** 2021-08-05

**Authors:** Kamran Waidha, Udi Zurgil, Efrat Ben-Zeev, Jacob Gopas, Saravanakumar Rajendran, Avi Golan-Goldhirsh

**Affiliations:** 1 DRDO-Defence Institute of High Altitude Research (DIHAR), Leh, Ladakh UT-194101, India; kamranwaidha1@gmail.com; 2 The Jacob Blaustein Institutes for Desert Research (BIDR), French Associates Institute for Agriculture and Biotechnology of Drylands, Sede Boqer Campus, Ben-Gurion University of the Negev, Sede Boqer 8499000, Israel; udizur@gmail.com; 3 Nancy and Stephen Grand Israel National Center for Personalized Medicine, The Department of Life Sciences Core Facilities, Weizmann Institute of Science, Rehovot 76100, Israel; efrat.benzeev@weizmann.ac.il; 4 The Shraga Segal Department of Microbiology, Immunology and Genetics Faculty of Health Sciences, Ben-Gurion University of the Negev, Beer Sheva 8410501, Israel; jacob@bgu.ac.il; 5 Department of Oncology, Soroka University Medical Center, Beer Sheva 8410501, Israel; 6 Chemistry Division, SAS, Vellore Institute of Technology Chennai Campus, Chennai 600127, India

**Keywords:** cysteine proteases, Cathepsin, 6,6′-dihydroxythiobinupharidine, *Nuphar*, M^pro^, SARS-CoV-2, covalent docking, molecular dynamic simulation

## Abstract

The specificity of inhibition by 6,6′-dihydroxythiobinupharidine (DTBN) on cysteine proteases was demonstrated in this work. There were differences in the extent of inhibition, reflecting active site structural-steric and biochemical differences. Cathepsin S (IC_50_ = 3.2 μM) was most sensitive to inhibition by DTBN compared to Cathepsin B, L and papain (IC_50_ = 1359.4, 13.2 and 70.4 μM respectively). DTBN is inactive for the inhibition of M^pro^ of SARS-CoV-2. Docking simulations suggested a mechanism of interaction that was further supported by the biochemical results. In the docking results, it was shown that the cysteine sulphur of Cathepsin S, L and B was in close proximity to the DTBN thiaspirane ring, potentially forming the necessary conditions for a nucleophilic attack to form a disulfide bond. Covalent docking and molecular dynamic simulations were performed to validate disulfide bond formation and to determine the stability of Cathepsins-DTBN complexes, respectively. The lack of reactivity of DTBN against SARS-CoV-2 M^pro^ was attributed to a mismatch of the binding conformation of DTBN to the catalytic binding site of M^pro^. Thus, gradations in reactivity among the tested Cathepsins may be conducive for a mechanism-based search for derivatives of nupharidine against COVID-19. This could be an alternative strategy to the large-scale screening of electrophilic inhibitors.

## 1. Introduction

**Figure 1:**
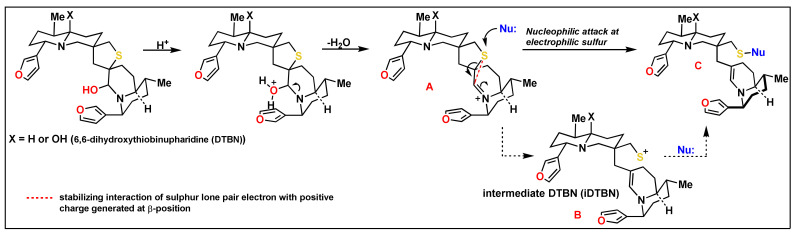
Hypothetical mechanism of interaction between the electrophilic sulfur of the nupharidine and a nucleophilic cysteine sulphur at the active site of the cysteine protease [19].

The yellow water lily, *Nuphar lutea* (L.) Sm. (Nymphaeaceae) contains a unique family of bioactive sesquiterpene thioalkaloids, nupharidines [1,2,3,4,5], whose biological therapeutic potential has been little studied to date. Recent reports from various laboratories, including ours, on the medicinal properties of *Nuphar* extracts, have indicated the potential therapeutic uses for these compounds as follows: anti-inflammatory [6], anti-bacterial [7,8], against pathogenic fungi [9], against the malaria, leishmania and trypanosome parasites [10,11,12], against cancer [3,7,13] and anti-viral activity against the measles virus [14]. Recently, our group demonstrated the bioactivity of 6,6′-dihydroxythiobinupharidine (DTBN) against PKC isoforms [15]. Here, we report, for the first time, a strong inhibitory activity of cysteine proteases by the 6,6′-dihydroxythiobinupharidine (DTBN). This finding could potentially increase understanding the mechanism of action of nupharidines and the design of site-specific drugs. Proteases are widely distributed in nature, bacteria, plant and human, and play central roles in many physiological and biological processes that are essential for life. Among the cysteine proteases, we have focused on the Cathepsins (CTSs), B, L and S [16], a family of proteases expressed in all living organisms. In humans, CTSs comprise 15 proteolytic enzymes that are classified into three distinct groups based on the key amino acids within their active site. Although CTSs are mainly localized in the lysosomes, where the acidic environment facilitates their proteolytic activity, they are also found in the cytoplasm, nucleus, and extracellular space, where they participate in extracellular matrix (ECM) protein degradation, cell signaling, protein-processing, and trafficking through the plasma and nuclear membranes and between intracellular organelles [16]. Abnormal expression and/or activity of CTSs has been associated with a variety of human diseases, including inflammatory and cardiovascular diseases, neurodegenerative disorders, diabetes, obesity, cancer, kidney dysfunction, viral diseases and many others. The activity of CTSs is regulated by interactions with their endogenous protein inhibitors: the cystatins, thyropins and some of the serpins [17]. Interest in small-molecule inhibitors of CTSs has been a research objective in recent years [18]. Most of the reported inhibitors are synthetic compounds. The DTBN is a new addition to this family of compounds and is unique as it is derived from a natural source, a plant; therefore, it is potentially more compatible with the cellular environment than a synthetic derivative. Furthermore, it has a unique structure and potential chemical reactivities as a sesquiterpene thio-alkaloid. Its pleotropic activity, as indicated above, shows its therapeutic potential. Exploration of its potential medicinal uses and understanding its mechanism of action is only a recent area of study [18]. The working mechanistic hypothesis for the effect of DTBN on proteases is that the electrophilic sulfur atom of the thiaspirane pharmacophore targets a nucleophilic cysteine at the active site of cysteine proteases (Figure 1) [19]. Thus, we tested the inhibitory effect of DTBN on various proteases in vitro and made molecular docking simulations in several proteases, in order to verify this potential interaction. In addition, we investigated the working mechanistic hypothesis of DTBN on various cysteine proteases through a series of molecular docking simulations.

In view of the current worldwide COVID-19 pandemic, it was challenging to test the effect of DTBN on the cysteine protease, M^pro^, of the new coronavirus, SARS-CoV-2 [20]. It is a large enveloped, positive sense, single-stranded RNA betacoronavirus. The viral RNA encodes two open reading frames that, through ribosome frame-shifting, generate two polyproteins [21]. These polyproteins produce most of the proteins of the replicase–transcriptase complex [22]. The polyproteins are processed by two viral cysteine proteases: a Papain-like protease (PLpro), which cleaves three sites, releasing non-structural proteins nsp 1–3, and a 3CL protease, also referred to as the main protease (M^pro^), (EC 3.4.22.69), that cleaves at 11 sites to release non-structural proteins (nsp 4–16), which play a key role in the infectivity of the virus. We tested the DTBN against this protease. To do so, we used the COVID Moonshot initiative (https://postera.ai/covid and https://www.nature.com/articles/s41557-020-0496-2 [23]), which assayed the inhibitory effect of the DTBN against SARS-CoV-2 M^pro^ [23]. Our strategy yielded a more comprehensive view of the mode of action and provided evidence for a plausible pleiotropic effect exhibited by DTBN. Insight into the structure–function relationship that leads to the high inhibitory selectivity among cysteine proteases could form a foundation for the development of new therapeutics against various diseases. We trust that DTBN is an important addition to the plethora of small-molecule inhibitors of cysteine proteases.

## 2. Results and Discussion

### 2.1. Computaitonal Analyses

Nupharidines are a class of terpene alkaloid known to exhibit antimetastatic activity [2], high cytotoxic activity against B16F10, U937 and HT 1080 cell lines [24], and inhibit human type II topoisomerase [25] and B16 melanoma cells [24]. The pleotropic effect of nupharidines was ascribed to electrophilic sulphur of thiaspirane, which targets nucleophilic sulphur at the active site of proteases. Since cathepsins belong to a family of proteases, we anticipated that 6,6′-dihydroxythiobinupharidine (DTBN) may exhibit an inhibitory action via the hypothesis proposed above. To better understand the mode of action of DTBN and the role active site cysteine plays in Cathepsin affinity, a series of molecular docking simulations and molecular dynamics simulations were initially performed to predict the binding mode of DTBN when docked to three types of Cathepsin protein (B, L and S) and SARS-CoV-2 M^pro^. This was further validated by in vitro study.

#### 2.1.1. Molecular Docking Analysis

DTBN was docked to the active site of Cathepsins B, L, S and SARS-CoV-2 M^pro^. Binding energy metrics revealed that DTBN possess low binding affinity against Cathepsin B, L and SARS-CoV-2 M^pro^, whereas it has a high binding affinity towards Cathepsin S.

**Figure 2:**
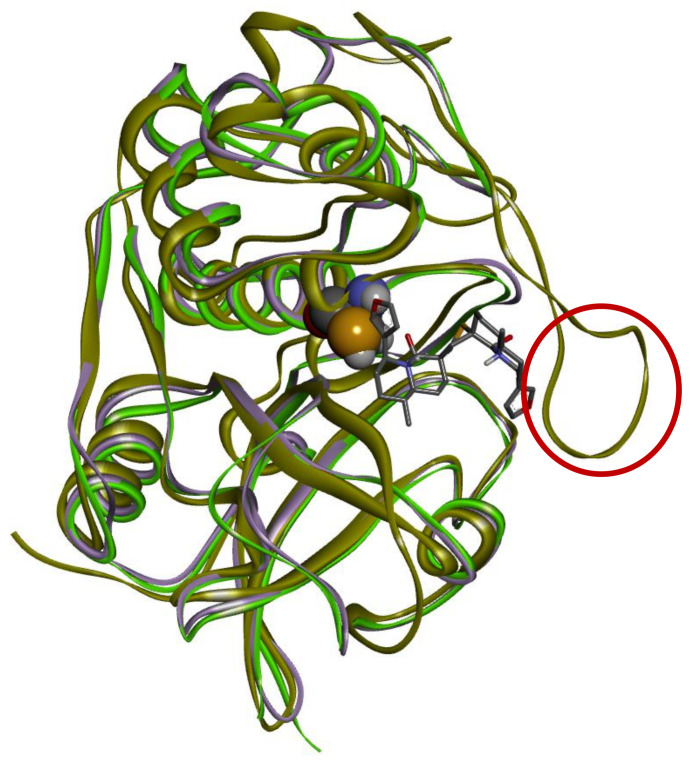
Superimposition of Cathepsin S (purple ribbon), Cathepsin L (green ribbon) and Cathepsin B (gold ribbon). Cathepsin B loop is indicated by a red circle. Docking of DTBN (grey lines) to Cathepsin B.

**Figure 3:**
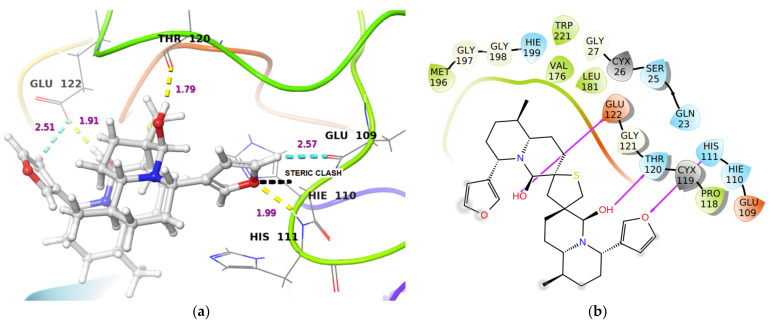
(**a**) 3-D representation of DTBN docked to Cathepsin B. H-bond interactions are displayed in yellow dotted lines, C-H…O hydrogen bonds are displayed in cyan blue dotted lines, steric clash is displayed in black dotted line. (**b**) 2-D Ligand interaction diagram of DTBN docked to Cathepsin B.

An analysis of molecular docking of DTBN with Cathepsin B, which shows the lowest binding energy (−42.57 Kcal/mol) of the Cathepsins, revealed that the 20 amino acids loop, which is missing in both Cathepsin S and L, is in close proximity to the binding site gorge (Figure 2). The loop creates a wall-like structure, blocking one side of the binding site and reducing the binding site volume. This leads to a steric clash between amino acid residue, the His110 present in the concluding loop and the furan ring of DTBN (Figure 3a), which might contribute to its lower binding energy.

However, at the active site of Cathepsin B, DTBN was stabilized by a hydrogen bonding interaction between -O of the furan ring in DTBN with -NH of His111 at the protein backbone (Table 1). In addition, the DTBN was stabilized by two other hydrogen bonds (i) between the hydroxy group of DTBN and -C(O) of Thr120 at protein backbone, (ii) between the hydroxy group of DTBN and side chain carboxylic -OH of Glu122 (Table 1). This gain in interactions may contribute to the final docking score (Figure 3). In comparison, the Cathepsin B inhibitor at the same active site is stabilized by three hydrogen bond interactions, ((i) between the side chain carboxylic -OH of Asp69 and carboxylic hydrogen of the inhibitor ((O)C-O**^…^**HO-C(O), 1.74 Å), (ii) between amide -NH of Asp69 and carboxylic -C(O) of the inhibitor (C(O)NH**^…^**(O)C-OH, 2.30 Å), (iii) between amide -NH of Gln23 and -NH-C(O) oxygen of the inhibitor (NH**^…^**(O)C-NH, 1.97 Å)), and by four aromatic C-H**^…^**O hydrogen bonds.

**Figure 4:**
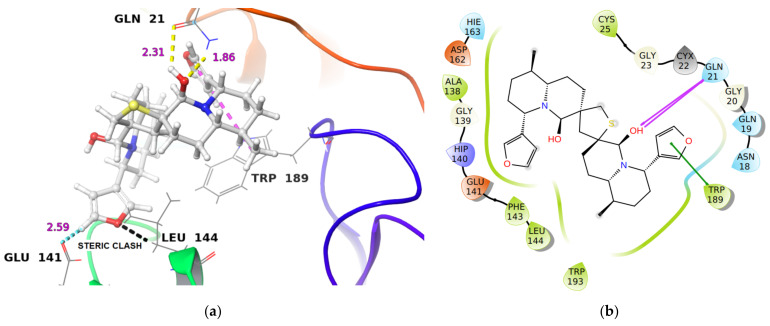
(**a**) 3-D representation of DTBN docked to Cathepsin L. H-bond interactions are displayed with yellow dotted lines, C-H**^…^**O hydrogen bonds are displayed with cyan blue dotted lines, π**^…^**π stacking interaction is displayed with pink dotted line, steric clash is displayed with black dotted line. (**b**) 2-D Ligand interaction diagram of DTBN docked to Cathepsin L.

Similarly, DTBN at the active site of Cathepsin L is stabilized by two hydrogen bond interactions (i) between hydroxy oxygen of DTBN and -NH of Gln21 at the protein back bone, and (ii) between hydroxy hydrogen of DTBN and -C(O) of Gln21 at the protein back bone. In addition, the DTBN was stabilized by the π**^…^**π stacking interaction between the furan ring of DTBN and indole ring of Trp189 (Figure 4). However, DTBN experiences a steric clash with amino acid residues at the active site. A steric clash is observed between the furan ring in DTBN and amino acid residue, Leu144 (Figure 4a), which might contribute to its low binding energy. In comparison, the Cathepsin L inhibitor at the same active site is stabilized by a hydrogen bond interaction (Asp162C(O)**^…^**HN of the inhibitor (1.72 Å)) and by three aromatic C-H**^…^**O hydrogen bond interactions. These stabilizing interactions likely contributed to its high binding energy and reactivity.

**Figure 5:**
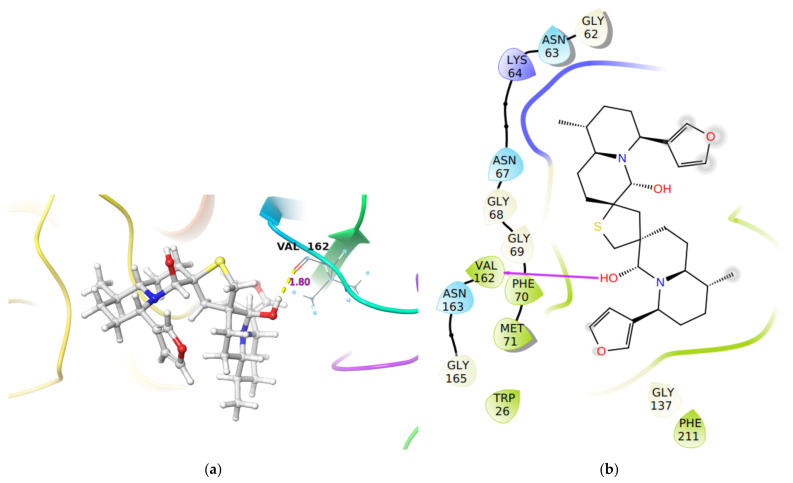
(**a**) 3-D representation of DTBN docked to Cathepsin S. H-bond interactions are displayed in yellow dotted lines, (**b**) 2-D Ligand interaction diagram of DTBN docked to Cathepsin S.

An analysis of the docking results of DTBN with Cathepsin S revealed that the ligand, DTBN, is stabilized by one hydrogen bond interaction at the active site (Figure 5). A hydrogen bond is observed between the hydroxy group of DTBN and -C(O) of Val162 at the protein backbone. Unlike Cathepsins B and L, in Cathepsin S there is no steric clash observed between protein and ligand, probably because the absence of a destabilizing interaction resulted in the observed high binding energy (Table 1). In comparison, the Cathepsin S inhibitor at the active site is stabilized by a hydrogen bond interaction between the side chain amide -NH of Asn161 and cyano group of the inhibitor ((O)C-NH_2_**^…^**NC, (2.02 Å)).

The molecular docking studies shown above predicted that the selectivity and specificity of the active site amino acids surrounding the cysteine would affect the binding site gorge size and shape. The active site is sandwiched between two β-barrel-shape polypeptides in the cysteine proteases, and the shape of the pace between them is important when fitting the inhibitor to the site, and for its effective inhibition. A profound difference is seen between Cathepsin B and the other tested cathepsins, where a loop of 20 amino acids is located in close proximity to the binding site, which may hinder the structural fitting of DTBN to the active site.

**Figure 6:**
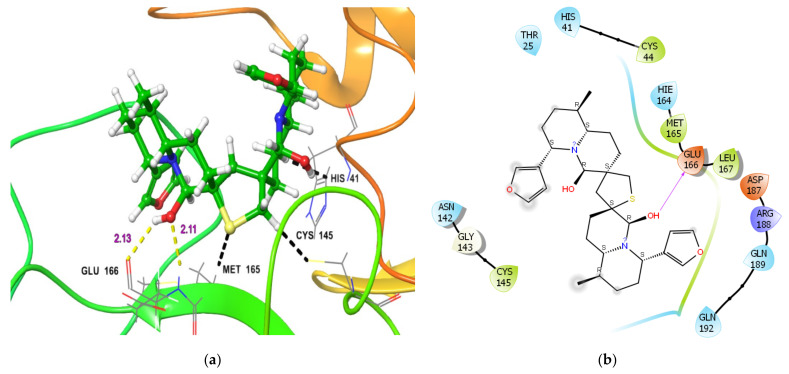
(**a**) 3-D representation of DTBN docked to M^pro^. H-bond interactions are displayed in yellow dotted lines, black dotted Line represent bad steric contacts/steric clashes, (**b**) 2-D Ligand interaction diagram of DTBN docked to M^pro^.

The main Protease (M^pro^) of SARS-CoV-2 in the active state exists as a homodimer. The homodimer is essential for the catalytic activity of M^pro^ and in shaping the S1 pocket of the substrate-binding site. The active site of M^pro^ features a cysteine amino acid residue, similar to the Cathepsins active site [30,31,32]. Therefore, we performed in silico docking analysis of DTBN to the substrate binding cleft (between Domain-1 and II) of M^pro^. Docking analysis revealed that the ligand, DTBN, is in close proximity to Cys145. At the active site (S1 subsite), a hydroxy group proximal to the tetrahydrothiophene ring of DTBN is hydrogen bonded with -NH hydrogen of Glu166 (2.13 Å) (Figure 6). Similarly, the hydroxy hydrogen is hydrogen-bonded with carbonyl oxygen of the α-carboxylic acid of Glu166. However, the binding conformation of DTBN leads to three steric clashes, (i) between the tetrahydrothiophene ring of nupharidine and Cys145 sulphur, (ii) between the quinolizidine ring of nupharidine and imidazole ring of His41 and (iii) between tetrahydrothiophene sulfur in DTBN and the OH group of Met165. These destabilizing interactions likely contributed to the observed low binding energy (−35.87 kcal/mol). In comparison, the M^pro^ inhibitor at the active active site is stabilized by 8 hydrogen bond interactions between co-crystalized inhibitor and amino acid residues, Glu166, His164, His163, Phe140, Gly143, Cys145, His41. The interactions of the co-crytalized inhibitor post docking were found to be similar to the crystal structure interaction, thus validating our docking protocol with deviation of RMSD 0.40 Å.

In M^pro^, although DTBN is in close proximity to Cys145, the binding conformation of DTBN led to three steric clashes, which restricted effective access to M^pro^ catalytic site. Thus, a mismatch of the binding conformation of DTBN with the M^pro^ active site and associated steric clashes with important amino acid residues (catalytic dyad Cys145 and His41) of the active site contributed to its low binding energy (−35.87 Kcal/mol). Therefore, we may observe a low inhibitory effect against M^pro^.

The above in silico molecular docking results show that DTBN experiences a steric clash at the active site of Cathepsins B, L and M^pro^, whereas, at the active site of Cathepsin S, DTBN did not experience any such steric clashes. Binding energy metrics and the interactions at the active site indicate that DTBN has a relatively higher binding affinity towards Cathepsin S in comparison to Cathepsins B, L and M^pro^. In brief, DTBN may form a relatively more stable complex with Cathepsin S than with Cathepsin B, L and M^pro^. This was further validated by molecular dynamic (MD) simulation.

#### 2.1.2. Covalent Docking Analysis

In order to evaluate the possible working mechanistic hypothesis, we performed a covalent docking of DTBN with Cathepsins of interest and M^pro^.

For covalent docking, possible intermediate DTBN (iDTBN) was generated from DTBN (Figure 1), docked to the active site of Cathepsins B, L and S and evaluated for potential interactions with nucleophilic cysteine sulphur at the active site. A disulfide (S-S) covalent bond formation was observed between the iDTBN and nucelophilic cysteine sulphur at the active sites. This indicates that DTBN may possibly inhibit Cathepsins via the proposed working mechanistic hypothesis. However, other possibilities cannot be ignored. In addition to the covalent linkage between iDTBN and Cathepsin, the iDTBN was stabilized by various weak interactions, which contribute to the observed binding energy (Table 2).

**Figure 7:**
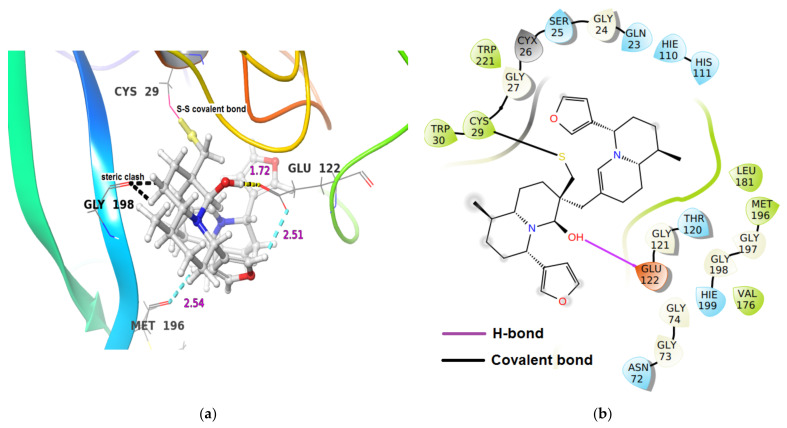
(**a**) 3-D representation of intermediate DTBN(iDTBN) docked to Cathepsin B. H-bond interaction is displayed by yellow dotted lines, C-H**^…^**O hydrogen bonds are displayed by cyan blue dotted lines, steric clashes are displayed by black dotted line, (**b**) 2-D Ligand interaction diagram of iDTBN docked to Cathepsin B.

In Cathepsin B-iDTBN covalent adduct, iDTBN was stabilized by one hydrogen bond interaction and two C-H**^…^**O aromatic hydrogen bond interactions (Table 2). However, iDTBN was destabilized by three sterich clashes: (i) between Glu122O-C(O)**^…^**H-O group of iDTBN, (ii) between Gly198O-C(O)**^…^**H-C of ring-opened thiaspirane in iDTBN, and (iii) Gly198O-C(O)**^…^**H-C of the quinolizidine ring in iDTBN). These steric clashes likely contributed to the low binding energy (Figure 7).

**Figure 8:**
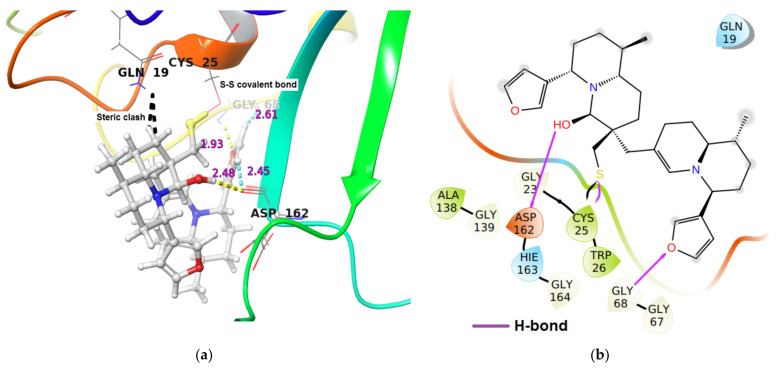
(**a**) 3-D representation of intermediate DTBN (iDTBN) docked to Cathepsin L. H-bond interaction is displayed by yellow dotted lines, C-H**^…^**O hydrogen bonds are displayed by cyan blue dotted lines, steric clashes are displayed by black dotted line, (**b**) 2-D Ligand interaction diagram of iDTBN docked to Cathepsin L.

Similarly, iDTBN was covalently docked to the active site of Cathepsin L and S. Similar to Cathepsin B, the iDTBN forms a covalent bond with nucleophilic cysteine sulphur at the active site of these enzymes. In the Cathepsin L-iDTBN covalent adduct, iDTBN was stabilized by two hydrogen bond interactions and by two C-H**^…^**O aromatic hydrogen bond interactions (Table 2). However, iDTBN was destabilized by two sterich clashes: (i) between Gln19 N-H…H-C of quinolizidine ring of iDTBN and (ii) between Gln19N-H**^…^**C-H of the quinolizidine ring of iDTBN). These steric interactions likely contributed to the observed binding energy (Figure 8).

**Figure 9:**
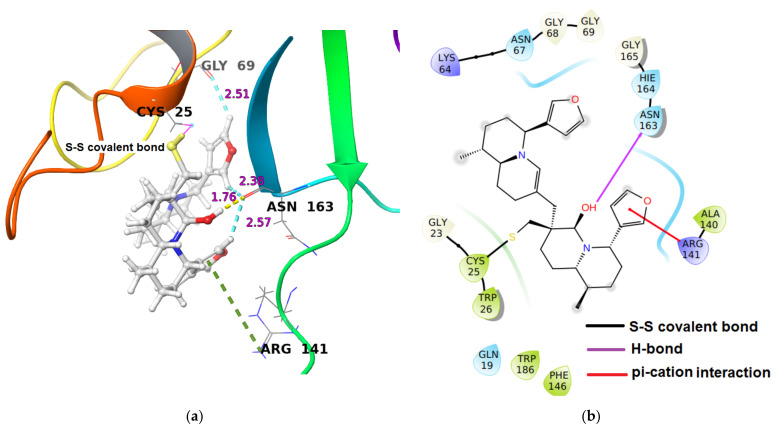
(**a**) 3-D representation of intermediate DTBN (iDTBN) docked to Cathepsin S. H-bond interaction is displayed by yellow dotted lines, C-H**^…^**O hydrogen bonds are displayed by cyan blue dotted lines, π**^…^**cation are displayed by green dotted line, (**b**) 2-D Ligand interaction diagram of iDTBN docked to Cathepsin S.

In the Cathepsin S-iDTBN covalent adduct, iDTBN was stabilized by one hydrogen bond interaction, three C-H**^…^**O aromatic hydrogen bond interactions and one π–cation interaction (Table 2). Unlike Cathepsin B and L-iDTBN covalent adduct, there no steric clash was observed in the Cathepsin S-iDTBN complexes. These stabilizing interactions likely contributed to the observed higher binding energy (Figure 9) as compared to Cathepsins B and L.

**Figure 10:**
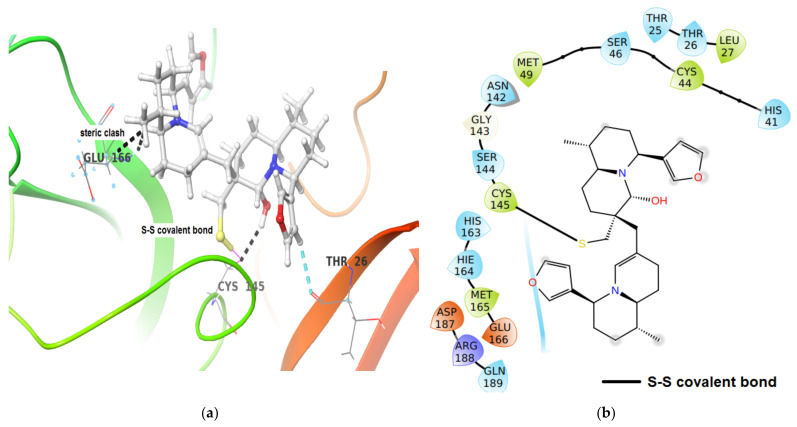
(**a**) 3-D representation of intermediate DTBN (iDTBN) docked to M^pro^. C-H**^…^**O hydrogen bonds are displayed in cyan blue dotted lines and steric clashes were displayed in black dotted lines, (**b**) 2-D Ligand interaction diagram of iDTBN docked to M^pro^.

Covalent docking of iDTBN to the active site of M^pro^ establishes a disulfide bond between iDTBN and sulphur of Cys145. In the M^pro^-iDTBN covalent adduct, iDTBN was stabilized by one C-H**^…^**O aromatic hydrogen bond (Figure 10), whereas, iDTBN was destabilized by three steric clashes: (i) between -S of amio acid residue, Cys145 of M^pro^ and hydroxy group in iDTBN, (ii) between –NH of amio acid residue, Glu166 of M^pro^ and -C-H of quinolizidine ring of iDTBN, and (iii) between side chain -CH_2_ of amino acid residue, Glu166 in M^pro^ and -C-H of quinolizidine ring of iDTBN. Although the M^pro^-iDTBN covalent adduct has a higher binding energy, steric clashes may destabilize the complex.

The above covalent docking results show that the elctrophilic sulphur of iDTBN and cysteine sulphur at the active site of Cathpesins and M^pro^ can potentially form a disulfide bond, which could be considered as supporting the proposed working mechanistic hypothesis. We presume that DTBN may mediate its inhibitory action via iDTBN. Binding energy metrics and interactions show that DTBN may exhibit a gradient inhbitory effect against Cathepsins B, L, S and M^pro^, with activity towards Cathepsin S.

#### 2.1.3. Molecular Dynamics (MD) Simulation

To determine the stability of docked poses of DTBN with Cathepsins and iDTBN-Cathepsins covalent adduct, and to find the stable interactions, a molecular dynamic (MD) simulation was performed. Both DTBN and iDTBN were subjected to 100 ns MD simulation with Cathepsins B, L and S and M^pro^.

#### Analysis of RMSD Value of Proteins and Ligands

**Figure 11:**
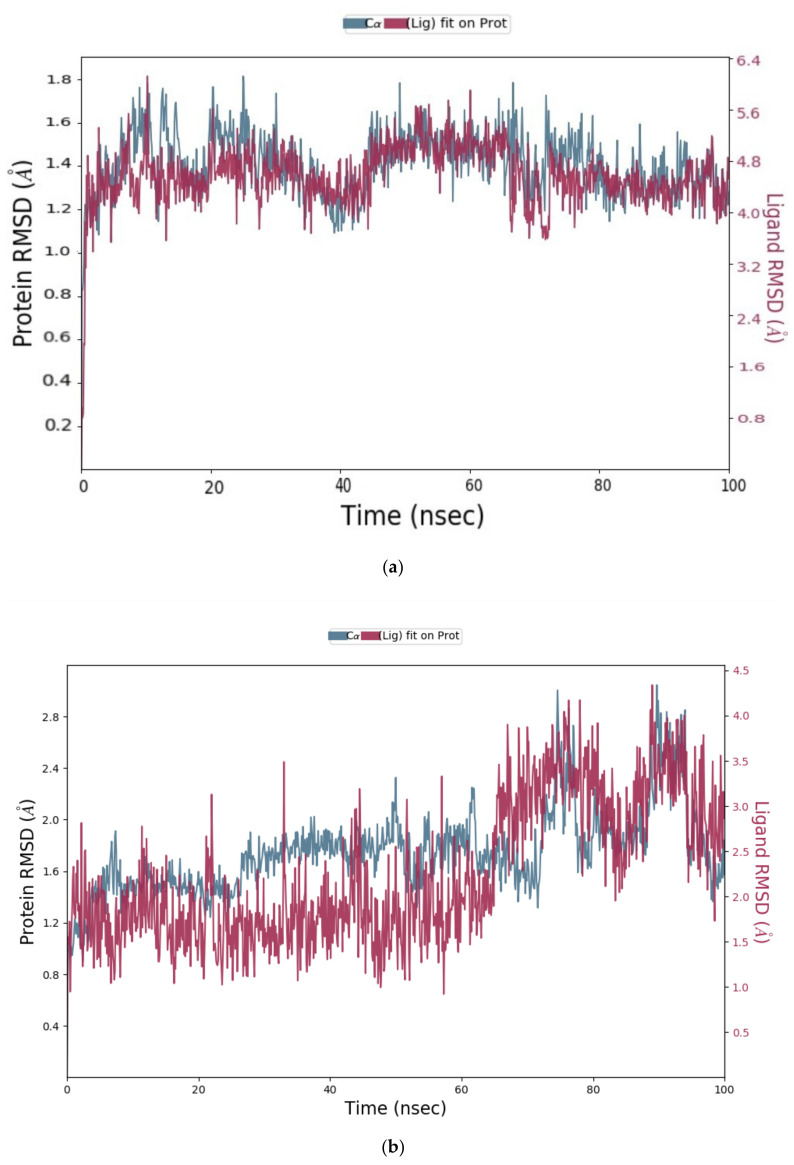
RMSD graph of (**a**) Cathepsin B-DTBN; (**b**) Cathepsin B-iDTBN covalent adduct.

**Figure 12:**
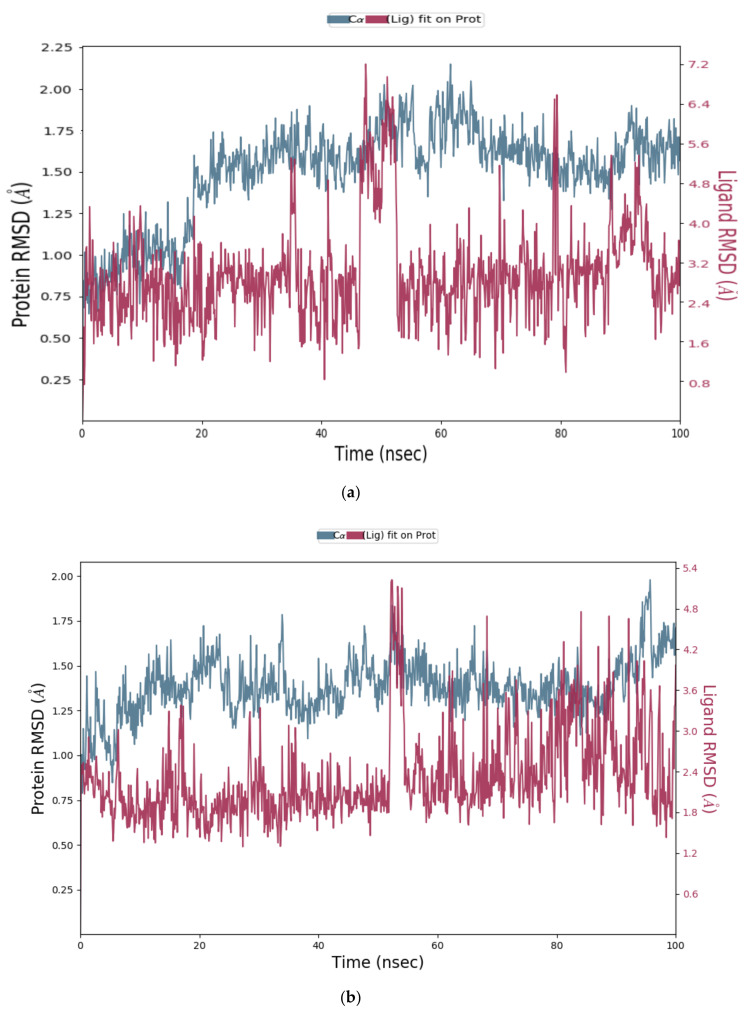
RMSD graph of (**a**) Cathepsin L-DTBN; (**b**) Cathepsin L-iDTBN covalent adduct.

**Figure 13:**
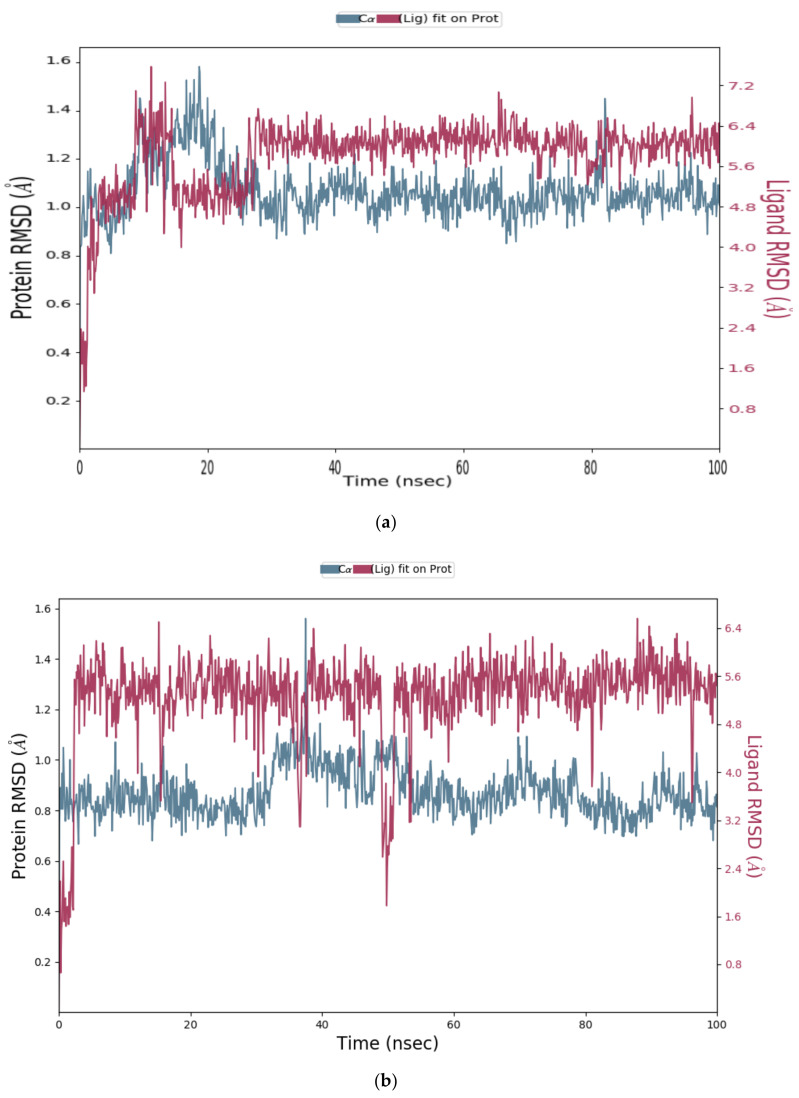
RMSD graph of (**a**) Cathepsin S-DTBN; (**b**) Cathepsin S-iDTBN covalent adduct.

**Figure 14:**
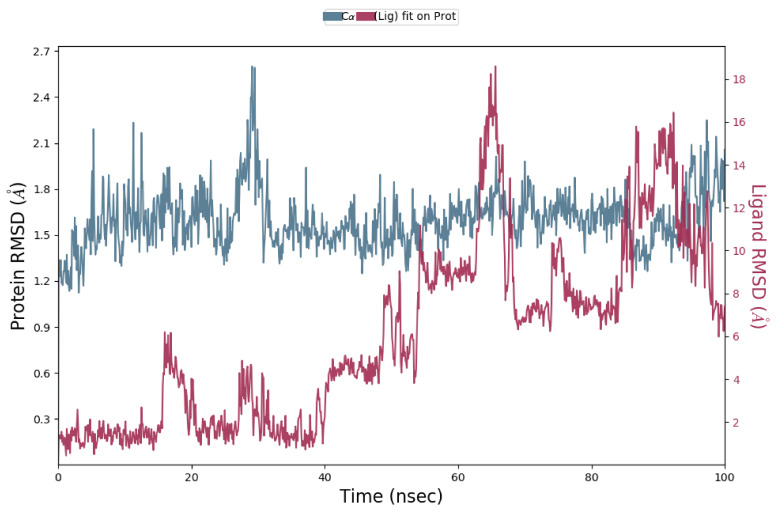
RMSD graph of M^pro^-DTBN.

The obtained MD simulation results were analysed using Cα and ligand RMSD values. The Root Mean Square Deviation (RMSD) value indicates the conformational changes in the ligand and protein from the initial structure during the simulation. For globular proteins, any RMSD value within 3 Å is acceptable [33]. Greater deviation from 3 Å implies that the protein in the protein–ligand complex is less stable due to the significant conformational changes that occurred during MD simulation. All seven protein–ligand complexes, Cathepsins B, L and S-DTBN, Cathepsins B, L and S-iDTBN and M^pro^-DTBN showed deviations in RMSD value well below 3 Å when compared to the initial frame throughout the trajectory (Figure 11, Figure 12, Figure 13 and Figure 14). This indicates that the Cathepsins and M^pro^ are stable in their respective protein–ligand complexes. Analysis of the ligand (DTBN and iDTBN) RMSD value against Cathepsins indicates that DTBN and iDTBN did not show a large deviation from their initial position during the MD run, signifying that their binding is stable. In contrast, DTBN shows a larger RMSD against M^pro^ during MD simulation (Figure 14). This indicates that it forms a less stable complex with M^pro^.

#### Analysis of RMSF Value of Proteins

Aside from RMSD value, the Root Mean Square Fluctuation (RMSF) values of proteins were also analysed. RMSF value is a measure of the flexibility of a protein residue during its interaction with the ligand in anMD run. Analysis of the RMSF value of Cathepsins (B, L and S) in Cathepsins-DTBN and iDTBN complexes shows that the RMSF value of all the proteins fall within 3.8 Å. This indicates that these proteins are stable while complexed with the ligand at the active site (Figures S1–S4 in the Supplementary Materials).

#### Molecular Interactions Observed during MD Simulations

The protein–ligand interactions of Cathepsins-DTBN, and M^pro^-DTBN and Cathepsins-iDTBN were continuously monitored throughout the simulation. The molecular interactions observed during MD simulations were captured and shown in Figures S5–S8 in the Supplementary Materials. These interactions were categorised into four types: hydrogen bond, hydrophobic, ionic and water bridges. The interactions of DTBN and iDTBN with specific amino acid residues of Cathepsins and M^pro^ during the MD run are shown in Figures S5–S8 in the Supplementary Materials.

#### Molecular Interactions That Exist above 30% of Minimum Contact Strength

*In Cathepsin B*

An MD simulation of Cathepsin B–DTBN shows two persistent hydrogen bond interactions above 30% of minimum contact strength. The hydroxy oxygen and hydrogen of DTBN were involved in the hydrogen bond with amino acid residue Gly198 at the active site (Figure S15a in the Supplementary Materials). However, in the Cathepsin B–iDTBN covalent adduct, no such interactions exceeded 30% of the minimum contact strength (Figure S15b in the Supplementary Materials).

*In Cathepsin L*

In Cathepsin L–DTBN MD simulation, there was one π**^…^**π stacking interaction that exceeded 30% of the minimum contact strength. The π**^…^**π stacking interaction was observed between the furan ring of DTBN and phenyl ring of amino acid residue, Trp189 (Figure S16a in the Supplementary Materials). In the Cathepsin L–iDTBN covalent adduct, one hydrogen bond was found to persistently have 70% contact strength (Figure S16b in the Supplementary Materials), indicating a strong interaction between Cathepsin L–iDTBN covalent adduct.

*In Cathepsin S*

At the active site of Cathepsin S, DTBN was stabilized by a prominent H-bond interaction (71%) between the amino acid residues Val162 and hydroxy group of DTBN, and by a π**^…^**π stacking interaction (36%) between the phenyl group of amino acid residues Phe211 and furan ring of DTBN (Figure S17a in the Supplementary Materials). At the active site of the S-iDTBN complex, iDTBN was stabilized by two interactions: (i) water bridged H-bond interaction (64%) between amino acid residues Asn163, water and the hydroxy group of iDTBN, and (ii) π**^…^**π stacking interaction (53%) between the phenyl group of amino acid residues Phe70 and furan ring of iDTBN (Figure S17b in the Supplementary Materials).

*In Mpro*

During the MD run of M^pro^-DTBN, DTBN was stabilized by a hydrogen bond interaction between the hydroxy group in DTBN and carbonyl oxygen α-carboxylic acid of amino acid residue, Glu166 which exceeded 40% of the minimum contact strength (Figure S18 in the Supplementary Materials).

The above MD simulation results suggest that DTBN and iDTBN form a stable complex with Cathepsins B, L and S. An analysis of the existing interactions between protein–ligand complexes during the MD run show that DTBN and iDTBN can form a more stable complex with Cathepsin S in comparison with Cathepsins B and L. On the other hand, the RMSD of DTBN and molecular interactions between the M^pro^-DTBN complex in MD simulation show that DTBN binds less stably to M^pro^.

In the docking results, it was shown that the cysteine sulphurs of M^pro^ and Cathepsin B, S and L are in close proximity to the nupharidine thiophene ring, potentially creating the conditions for a nucleophilic attack. The results presented here are compatible with the plausible mechanism of action of the 6,6′-dihydroxythiobinupharidine (DTBN), with the cysteine proteases proposed in Figure 1. This provides further support to the demonstration by Shenvi’s group that the sulfur atom of the Nuphar thiaspirane iminium pharmacophore reacts as an electrophile with nucleophilic thiols at ambient temperature. In dihydroxy nupharidine dimers, although not in monohydroxydimers, this reactivity causes a rapid retrodimerization to electrophilic, unsaturated iminium ions, which was ascribed to retrodimerization and, potentially, a highly reactive covalent binder [19]. It was proposed that the sulfur electrophilicity in the iminium thiaspirane could lead to the design of new therapeutics based on the Nuphar pharmacophore and new tools for the selective capture of free thiols in a cellular setting [19].

The above in silico molecular docking analysis and MD simulation studies show the DTBN’s potential ability to inhibit Cathepsins (B, L and S) and M^pro^. However, the protein-ligand stability and molecular interactions show that DTBN may exhibit gradient inhibitory activity against Cathepsins and M^pro^, with better activity against Cathepsin S in comparison to Cathepsin B and L and the least inhibitory activity against M^pro^. To validate the obtained molecular docking and MD simulation results, an in vitro assay was conducted, and the results were compared.

### 2.2. In Vitro Analysis

#### Enzyme Activities

The inhibitory activity of DTBN against various proteases is shown in Table 3. There was a high specificity towards papain and cysteine proteases of the Cathepsins family B, L and S. However, there was no inhibition of calpain, which belongs to the family of calcium-dependent, non-lysosomal cysteine proteases, or towards trypsin, which is a serine protease and does not act against the SARS-CoV-2 M^pro^ protease. Within the Cathepsins, there was a significant gradation of DTBN inhibitory activity; while Cathepsin S was strongly inhibited in the low micromolar range, with an IC_50_ value of 3.2 µM, Cathepsins L was less inhibited, with an IC_50_ values of 13.2 µM. Cathepsin B was least inhibited with an IC_50_ value of 1359.4 µM (Table 3).

The specificity of inhibition by DTBN on cysteine proteases was demonstrated in this work. There were differences in the extent of inhibition, reflecting structural-steric and biochemical differences in the active site. Cathepsin S was most sensitive to inhibition by DTBN, compared to cathepsin B, L and papain. DTBN was inactive for the inhibition of SARS-CoV-2 M^pro^ and non-cysteine proteases, Trypsin and Calpain.

A series of molecular docking simulations suggested a mechanism of interaction that supported the biochemical results. The binding energy metrics and protein–ligand interactions of the docked poses of DTBN with Cathepsins and M^pro^ obtained from molecular docking studies clearly show that DTBN has a variable affinity towards M^pro^ and Cathepesins B, L and S, although belonging to the cysteine proteases family. The difference in affinity was attributed to structural-steric and biochemical differences in the active site. The proposed possible working mechanistic hypothesis for DTBN is that the electrophilic sulphur of DTBN targets nucleophilic cysteine sulphur at the active site of Cathepsins (B, Land S) or M^pro^ to form a disulphide bond. This was validated by a covalent docking study. The binding energy and molecular interaction suggested that DTBN has a stronger interaction with Cathepsin S in comparison with Cathepsins B, L and M^pro^. Further, a molecular dynamic simulation was performed to evaluate the stability of M^pro^-DTBN, the Cathepsins–DTBN complex, and their covalent adducts and the associated molecular interactions during the MD run. The stability of the protein–ligand complex and associated molecular interactions suggested that DTBN formed teh most stable complex with S, followed by Cathepsins L, B and, finally, M^pro^. The results obtained from molecular docking, covalent docking and molecular dynamic simulation studies revealed the potential ability of DTBN to inhibit Cathepsins B, L and S and Mpro. However, its activity may incline towards Cathepsin S in comparison to Cathepsins B, L and Mpro. This was validated by in vitro assay. The experimental results are in accordance with the predicted computational docking studies.

## 3. Materials and Methods

### 3.1. Materials

6,6′-dihydroxythiobinupharidine (DTBN) was purified from *Nuphar lutea* extracts and purchased from Sigma (St. Louis, MO, USA)/Merck (Darmstadt, Germany), cat. SMB00609. DTBN was dissolved in DMSO.

### 3.2. Computational Methodology

#### 3.2.1. Molecular Docking

Molecular docking was performed using Schrödinger Maestro Suite 2020-3 (Schrödinger, LLC, New York, NY, USA).

#### 3.2.2. Retrieval of Protein Crystal Structures

Three-dimensional crystal structures of Cathepsins (Cathepsin B (PDB ID: 1GMY), Cathepsin L (PDB ID: 2XU3), Cathepsin S (PDB ID: 3N3G) and Mpro (PDB: 6Y2F) proteins were retrieved from RCBS PDB database. All structures were optimized prior to docking using the Protein Preparation Wizard in Schrödinger Maestro Suite 2020 (Schrödinger Suite 2020-3 Protein Preparation Wizard; Epik, Schrödinger, LLC, New York, NY, USA, 2020; Impact, Schrödinger, LLC, New York, NY, USA, 2020; Prime, Schrödinger, LLC, New York, NY, USA, 2020.). Inconsistencies in the structure, such as missing hydrogen, incorrect bond orders and orientation of the different functional groups of amino acids, were rectified during the optimization process [34]. The prepared proteins were then used for molecular docking. The amino acid numbering is same as that of the crystal structure.

#### 3.2.3. Ligand Preparation

The structure of 6,6′-dihydroxythiobinupharidine ligand was prepared prior to docking using the LigPrep application in Schrödinger Maestro Suite 2020 (LigPrep, Schrödinger, LLC, New York, NY, USA, 2020-3). LigPrep was used to perform the 2-dimensional to 3-dimensional conversion of structures, correct improper bond distances and bond orders, and generate ionization states and energy minimization processes. This ligand structure prepared by LigPrep was then used for further studies.

#### 3.2.4. Molecular Docking and MM-GBSA Refinement

Molecular docking studies were performed using Schrödinger Maestro Suite 2020-3 (Schrödinger, LLC, New York, NY). DTBN was docked to the active site of Cathepsins B, L and S. Cathepsins B and L the center of the grid was generated against the centroid of previously known active site residue given in Table 4.

For Cathepsin S, the co-crystallized ligand was used as the centeroid of the grid. In Mpro, the crystal structure of the original ligand was removed from the complex and Cys145 residues were selected to function as the centroid of ligand binding site definition, as they play a crucial role in catalytic dyad. This was further used to generate the receptor grid with an internal ligand diameter midpoint box of the size 15 × 15 × 15 Å^3^. A post-docking simulation MM-GBSA refinement was carried out on the docked poses with a flexible residue distance of 5 Å.

The binding energy was calculated by the following formula:

ΔG = Σ(ΔG_Bind coulomb_ + ΔG_Bind covalent_ + ΔG_Bind H-bond_ + ΔG_Bind Lipo_ + ΔG_Bindpacking_ + ΔG_Bind Self cont_ + ΔG_Bind Solv GB_ + ΔG_Bind vdw_)

#### 3.2.5. Covalent Docking

To establish a covalent bond linkage between the protein and iDTBN (Figure 1) Schordinger’s CovDock application was used. For Cathepsin B, Cys29 was selected to act as a reactive residue. Similarly, for Cathepsin L and S, Cys25 was selected to act as a reactive residue in both cases. In M^pro^, Cys145 was selected to act as the reactive residue. The aforementioned cysteine residues were used to act as the center of the docking grid and docking ligands, with a length of less than 20 Å. The pose-prediction docking mode was utilized, and post-docking MMGBSA scoring was also performed.

#### 3.2.6. Molecular Dynamics (MD) Simulation

MD simulation was performed using a DESMOND module by Schordinger (Schordinger relase 2021). Through the system builder panel, DTBN and iDTBN with a protein complex were immersed in an orthorhombic box of TIP3P solvent model. The solvated system was neutralized using counter ions and physiological NaCl salt concentration of 0.15 M. OPLS4 force field was utilized. The simulation was 100 ns using NPT assemble class at a temperature of 300 K and atmospheric pressure of 1.013 bar.

### 3.3. In Vitro Methodology

#### Enzymes Assays

Enzyme inhibition assays were performed in triplicates using transparent 96-well plates for Calpain, Trypsin, Papain, Cathepsin L and Cathepsin B (167008; Nunc, Roskilde, Denmark), or black ones for Cathepsin S, (Greiner 96 Polystyrol GRE96fb), according to manufacturers’ instructions. Briefly, a reaction mixture of 100 µL contained: 45µL of enzyme in assay buffer, 5 µL of inhibitor diluted in DMSO. 50 µL substrate prepared in assay buffer according to manufacturers’ instructions. The inhibitor was serially diluted in DMSO to concentrations from 0.0075 µg/µL to 0.0000075 µg/µL. A total of 50 µL DMSO was added to the control sample. The enzyme substrate mix was added last, just before starting the fluorometric measurement. Data were collected for 1.5 h at 60 sec intervals.

Papain Latex (cat. No. p3125), Human Cathepsin B (cat.no. C0150), Trypsin and Calpain (cat. No. ab910119) and the substrate, casein-FITC, were obtained from Sigma-Aldrich. The assay was carried out in 0.3 M potassium chloride, 0.1 mM EDTA, and 3 mM DTT pH 6.5. Ex/Em = 485–535 nm. Human Cathepsins S (cat. No. 1183-cy) and L (cat.no. ab174030) and their substrates were Mca-RPKPVE-Nval-WRK (Dnp)-NH2 (ES002) and Ac-FR-AFC, respectively. They were obtained from ABCAM. Assay buffer for Human Cathepsin S was 50 mM NaOAc, 250 mM NaCl, 5 mM DTT pH 4.5. Ex/Em = 320–405 nm and, for Cathepsin L, the assay buffer was CTSL, Ex/Em = 400–505 nm.

The assay was conducted according to [35]. Compounds were seeded into assay-ready plates (Greiner 384 low volume 784900) using an Echo 555 acoustic dispenser and DMSO was back-filled for a uniform concentration in assay plates (maximum 1%). Reagents for M^pro^ assay were dispensed into the assay plate in 10 µL volumes for a total of 20 µL. Final reaction concentrations were 20 mM HEPES pH = 7.3, 1 mM TCEP, 50 mM NaCl, 0.01% Tween-20, 10% glycerol, 5 nM M^pro^ 375 nM fluorogenic peptide substrate ([5-FAM]-AVLQSGFR-[Lys(Dabcyl)]-K-amide). M^pro^ was pre-incubated for 15 min at room temperature with compound before the addition of substrate. Protease reaction was measured continuously in a BMG Pherastar FS with a 480/520 ex/em filter set. Data were mapped and normalized in the Genedata Screener.

## 4. Conclusions

Cathepsins, a family of proteases expressed in all living organisms, including humans, participate in protein degradation, cell signaling and protein-processing. Abnormal expression and/or activity of Cathepsins has been associated with a variety of human diseases, including cancer, inflammatory and cardiovascular diseases, neurodegenerative disorders, viral diseases, and many others. 6,6′-Dihydroxythiobinupharidine (DTBN), a bioactive sesquiterpene thioalkaloid, is present in yellow water lily, *Nuphar lutea* (L.) Sm. (Nymphaeaceae) was investigated as an inhibitor of Cathepsins and other proteases for comparative purposes. DTBN shows differences in the extent of Cathepsins inhibition, reflecting structural-steric and biochemical differences in the active site. Cathepsin S was most sensitive to inhibition by DTBN compared to Cathepsin B, L and papain. DTBN was inactive for the inhibition of SARS-CoV-2 M^pro^ of COVID-19 and non-cysteine proteases, Trypsin and Calpain. Docking simulations suggested a mechanism of interaction that was supported by the biochemical result. In the docking results, it was shown that the active site cysteine sulphur of Cathepsin S, L and B is in close proximity to the nupharidine tetrahydrothiophene ring, potentially creating the necessary conditions for a nucleophilic attack. Covalent docking of intermediate DTBN (iDTBN) to the active site of Cathepsins led to a disulfide bond between the electrophilic sulphur of iDTBN and nucleophilic cysteine sulphur of Cathepsins. This explains the potential of electrophilic sulphur in DTBN as active warhead that inhibits nucleophilic cysteine sulphur containing enzymes. Further, molecular dynamic (MD) simulation revealed the stability of protein–ligand complexes, where Cathepsin S formed the more stable complex with DTBN and iDTBN in comparison with Cathespsins B and L. The order of stability was in accordance with in vitro results, emphasizing the importance of the stability of the protein–ligand complex for inhibition. DTBN’s lack of reactivity against SARS-CoV-2 M^pro^ was attributed to a mismatch of binding conformation of DTBN to the catalytic binding site of SARS-CoV-2 M^pro^. Our results may be conducive for a mechanism-based search of derivatives of nupharidine against COVID-19. This could be an alternative strategy to the large-scale screening of electrophilic inhibitors.

## Figures and Tables

**Table 1:** DTBN binding energy * (ΔG) with Cathepsins and M^pro^.

	DTBN Binding Energy * ΔG (Kcal/mol)	Cathtepsin Inhibitor’s Binding Energy * ΔG (Kcal/mol)	Cathtepsin Inhibitor’s IC_50_ Values	H-Bond Interaction of Cathepsins-DTBN (Bond Length Å)
Cathepsin B	−42.57	−68.3	6.8 nM [26]	(i) His111 NH^…^O of furan ring in DTBN (1.99 Å) (ii) Thr120 C(O)^…^HO group in DTBN (1.79 Å) (iii) Glu122 (O)C-O^…^H-O group in DTBN (1.91 Å)
Cathepsin L	−42.59	−51.29	3.0 nM [27]	(i) Gln21 C(O)^…^HO group in DTBN (2.31 Å) (ii) Gln21 NH^…^OH group in DTBN (1.86 Å)
Cathepsin S	−44.3	−48.4	6.0 nM [28]	Val162 C(O)^…^H-O group in DTBN (1.80 Å)
M^pro^	−35.87	−76.14	0.67 μM [29]	(i) Glu166 NH^…^OH group in DTBN (2.13 Å) (ii) Glu166 O-C(O)^…^HO in DTBN (2.11 Å)

* post MMGBSA refinement.

**Table 2:** Covalent binding Energy * (ΔG), of iDTBN with Cathepsins (B, L and S) and M^pro^.

Enzyme	iDTBN Covalent Dock Binding Energy * ΔG (Kcal/mol)	Hydrogen Bond Interaction of iDTBN-Cathepsins and M^pro^ (Bond Length Å)
Cathepsin B	−18.57	(i) One hydrogen bond interaction Glu122O-C(O)^…^H-O group proximal to thiaspirane ring of DTBN (1.72 Å). (ii) two C-H^…^O aromatic hydrogen bond interactions (a) Glu122(O)C-O^…^H-C of furan ring in DTBN (2.51 Å) and (b) Met196C(O)^…^H-C of furan ring in DTBN (2.54 Å)
Cathepsin L	−28.9	(i) Two hydrogen bond interaction (a) Gly68N-H^…^O of furan oxygen of DTBN (1.93 Å) (b) Asp162C(O)^…^H-O of hydroxy group in DTBN (2.48). (ii) two C-H^…^O aromatic hydrogen bond interactions (a) Gly68C(O)^…^H-C of furan ring of DTBN (2.61 Å). (b) Asp162C(O)^…^H-C of furan ring of DTBN (2.45 Å)
Cathepsin S	−33.99	(i) One hydrogen bond interaction: Asn163O-C(O)^…^H-O of hydroxy group distal from thiaspirane ring in DTBN (1.76 Å), (ii) three C-H^…^O aromatic hydrogen bond interactions (a) Asn163C(O)^…^H-C of furan ring of DTBN (2.57 Å). (b) Asn163C(O)^…^H-C of furan ring of DTBN (2.38 Å). (c) Gly69C(O)^…^H-C of furan ring of DTBN (2.51 Å). (iii) one π^…^cation interaction: Arg141H-C=N-H^+^…π electron of furan ring in DTBN.
M^pro^	−46.53	Thr26C(O)^…^H-C of furan ring of DTBN (2.61 Å).

* post MMGBSA refinement.

**Table 3:** Inhibition of several proteases by DTBN.

Enzyme	IC_50_ (µM) (SD)
Human Cathepsin S	3.2 (0.3)
Human Cathepsin L	13.2 (0.6)
Human Cathepsin B	1359.4 (192.0)
Papain Latex	70.4 (0.6)
Trypsin *	n.i
Calpain *	n.i
SARS-CoV-2, M^pro^ **	n.i

Inhibition reactions were conducted in reaction mixtures of 100 µL, containing DTNB in the range of 10 µM and 1 mM. * Non-cysteine proteases. ** Tested at 20 and 50 μM of DTBN. n.i = no inhibition.

**Table 4:** Active site residues numbers for grid generation.

Cathepsin	Active Site Residue Number
Cathepsin B	23, 29, 75, 110, 199, 245
Cathepsin L	25, 19, 162, 161, 214, 69, 72, 61, 63, 68

## Data Availability

Not applicable.

## Supplementary Materials

The Supplementary Materials are available online.

## Sample Availability

Samples of the compound DTBN is available from the authors.
