# Supplementary material for: Inhibition of Cysteine Proteases by 6,6′-Dihydroxythiobinupharidine (DTBN) from Nuphar lutea
Source: Molecules. 2021 Aug 5;26(16):4743. doi: 10.3390/molecules26164743 (PMC8399019; doi:10.3390/molecules26164743)
Supplement: Supplementary file 1 [file molecules-26-04743-s001.zip › molecules-1249598-supplementary.pdf]

molecules

MDPI

Supplementary material

Inhibition of Cysteine Proteases by 6,6′-Dihydroxythiobinupharidine (DTBN) from *Nuphar lutea*

Kamran Waidha [1,†], Udi Zurgil [2,†], Efrat Ben-Zeev [3], Jacob Gopas [4,5], Saravanakumar Rajendran [6,*,†] and Avi Golan-Goldhirsh [2,*,†]

1 DRDO-Defence Institute of High Altitude Research (DIHAR), Leh, Ladakh UT-194101, India; kamranwaidha1@gmail.com
2 The Jacob Blaustein Institutes for Desert Research (BIDR), French Associates Institute for Agriculture and Biotechnology of Drylands, Sede Boqer Campus, Ben-Gurion University of the Negev, Sede Boqer 8499000, Israel; udizur@gmail.com (U.Z); avigolan@bgu.ac.il (A.G.-G.)
3 Nancy and Stephen Grand Israel National Center for Personalized Medicine, The Department of Life Sciences Core Facilities, Weizmann Institute of Science, Rehovot 76100, Israel; efrat.benzeev@weizmann.ac.il
4 The Shraga Segal Department of Microbiology, Immunology and Genetics Faculty of Health Sciences, Ben-Gurion University of the Negev, Beer-Sheva 8410501, Israel; jacob@bgu.ac.il
5 Department of Oncology, Soroka University Medical Center, Beer Sheva 8410501, Israel
6 Chemistry Division, SAS, Vellore Institute of Technology Chennai Campus, Chennai 600127, India
* Correspondence: sar.org@gmail.com (S.R.); avigolan@bgu.ac.il (A.G.-G.)
† Equal contributors.

Table of Contents

Figure S1: Cathepsin B-DTBN RMSF graph
Figure S2: Cathepsin B-iDTBN covalent dock RMSF graph
Figure S3: Cathepsin L-DTBN RMSF graph
Figure S4: Cathepsin L-iDTBN covalent dock RMSF graph
Figure S5: Cathepsin S-DTBN RMSF graph
Figure S6: Cathepsin S-iDTBN covalent dock RMSF graph
Figure S7: M^{pro}-DTBN RMSF graph
Figure S8: Represents the types of interactions in Cathepsin B–DTBN complex
Figure S9: Represents the types of interactions in Cathepsin B–iDTBN complex
Figure S10: Represents the types of interactions in Cathepsin L–DTBN complex
Figure S11: Represents the types of interactions in Cathepsin L–iDTBN complex
Figure S12: Represents the types of interactions in Cathepsin S–DTBN complex
Figure S13: Represents the types of interactions in Cathepsin S–iDTBN complex
Figure S14: Represents the types of interactions in M^{pro}–DTBN complex
Figure S15: 2-D Representation of various interaction between Cathepsin B–DTBN and iDTBN in MD simulation
Figure S16: 2-D Representation of various interaction between Cathepsin L–DTBN and iDTBN in MD simulation
Figure S17: 2-D Representation of various interaction between Cathepsin S–DTBN and iDTBN in MD simulation
Figure S18: 2-D Representation of various interaction between M^{pro}–DTBN in MD simulation

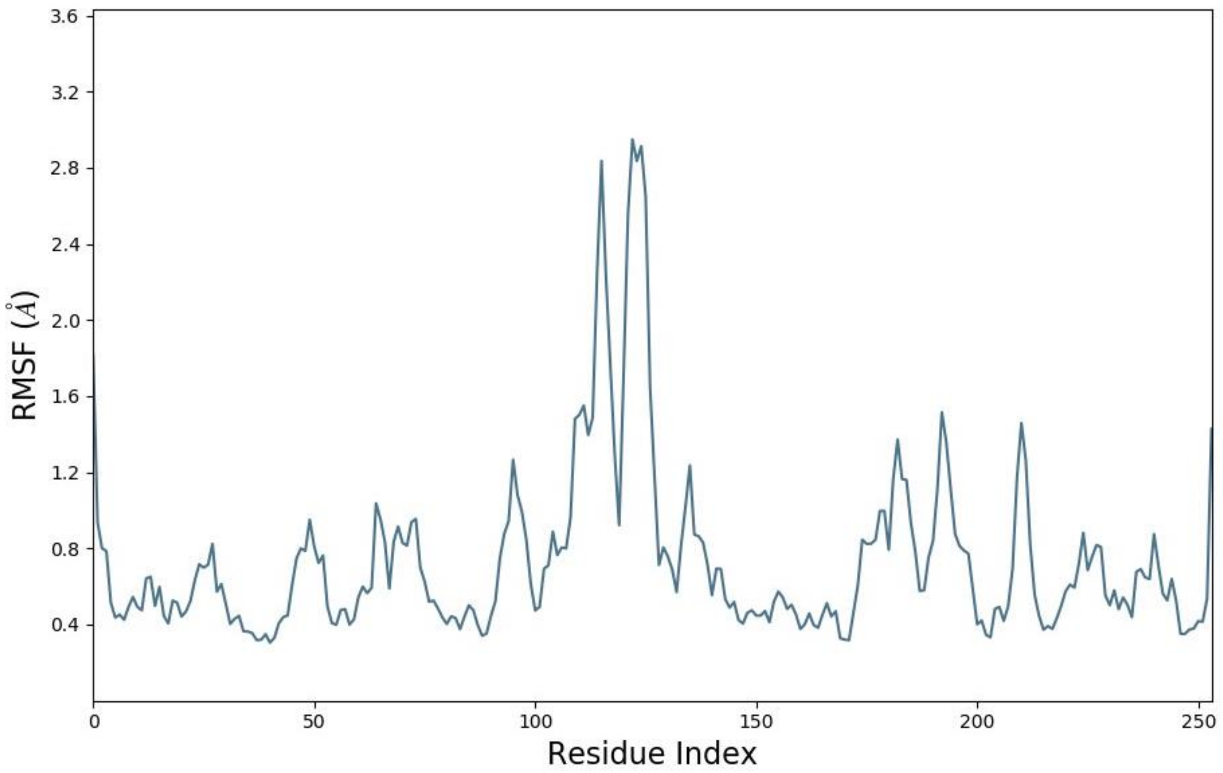

Figure S1: Cathepsin B-DTBN RMSF graph

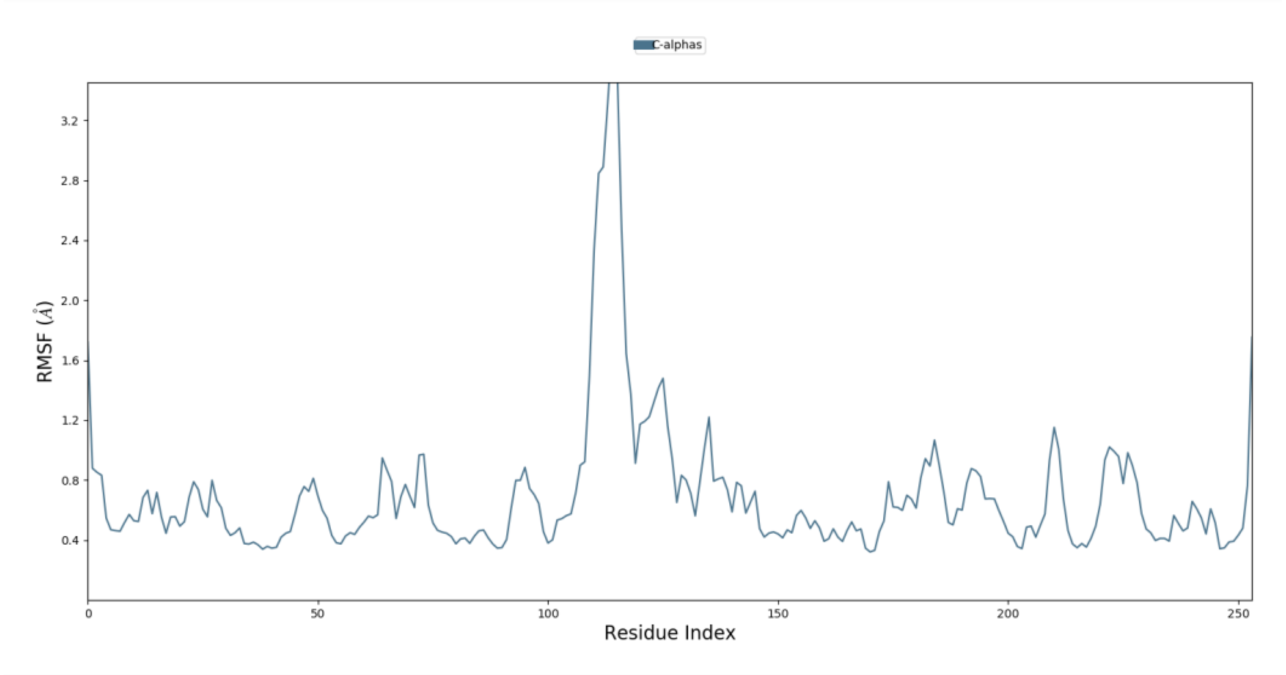

Figure S2: Cathepsin B-iDTBN covalent dock RMSF graph

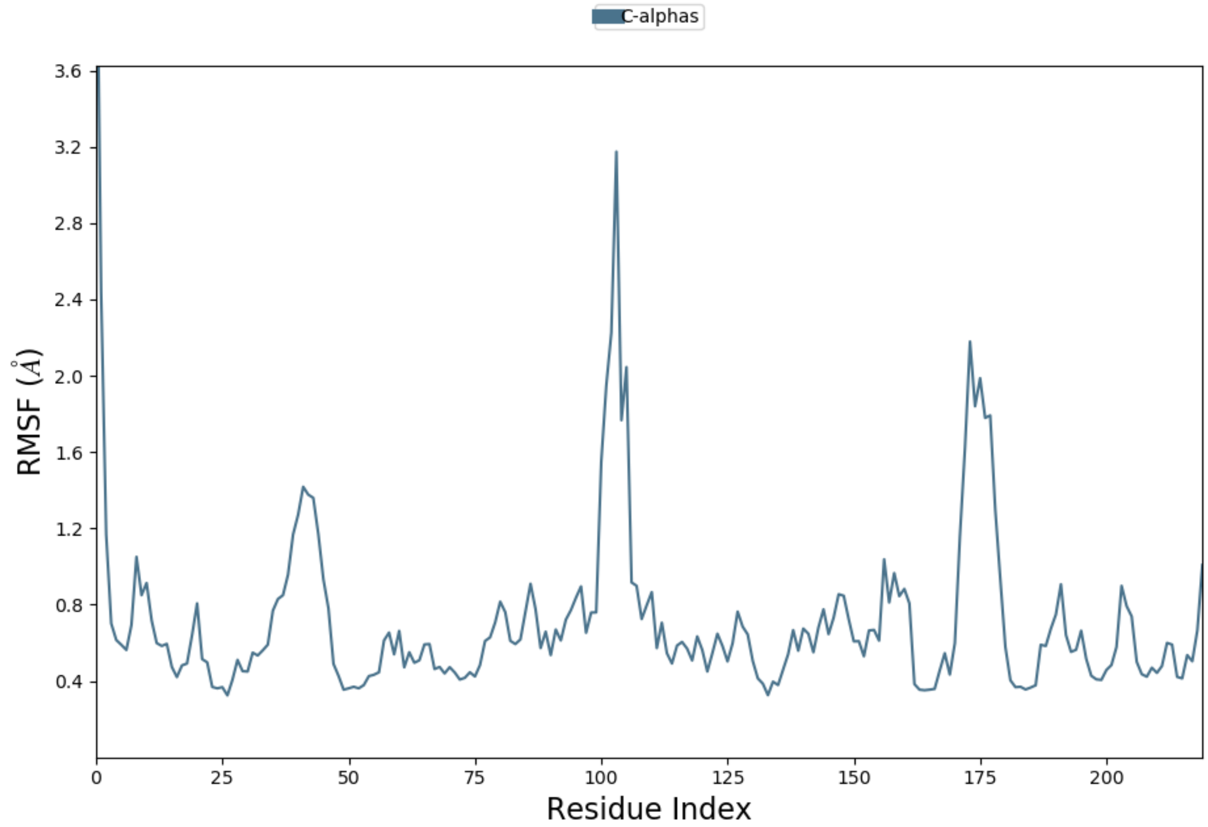

Figure S3: Cathepsin L-DTBN RMSF graph

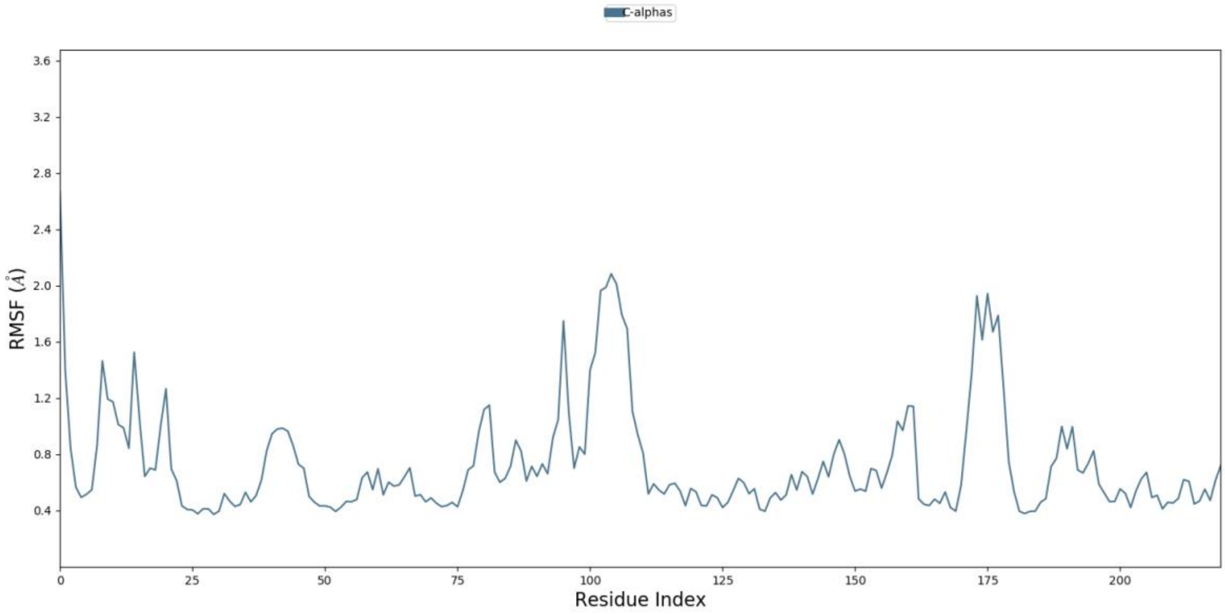

Figure S4: Cathepsin L-iDTBN covalent dock RMSF graph

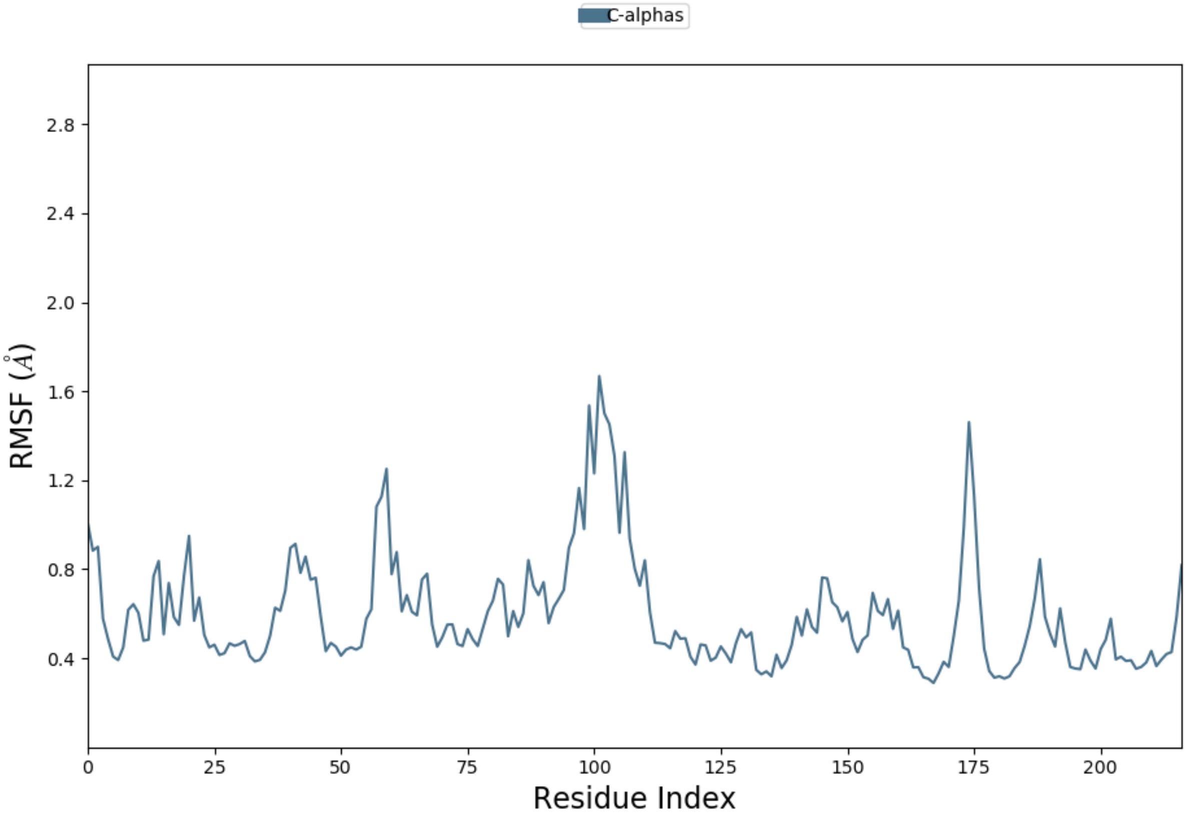

Figure S5: Cathepsin S-DTBN RMSF graph

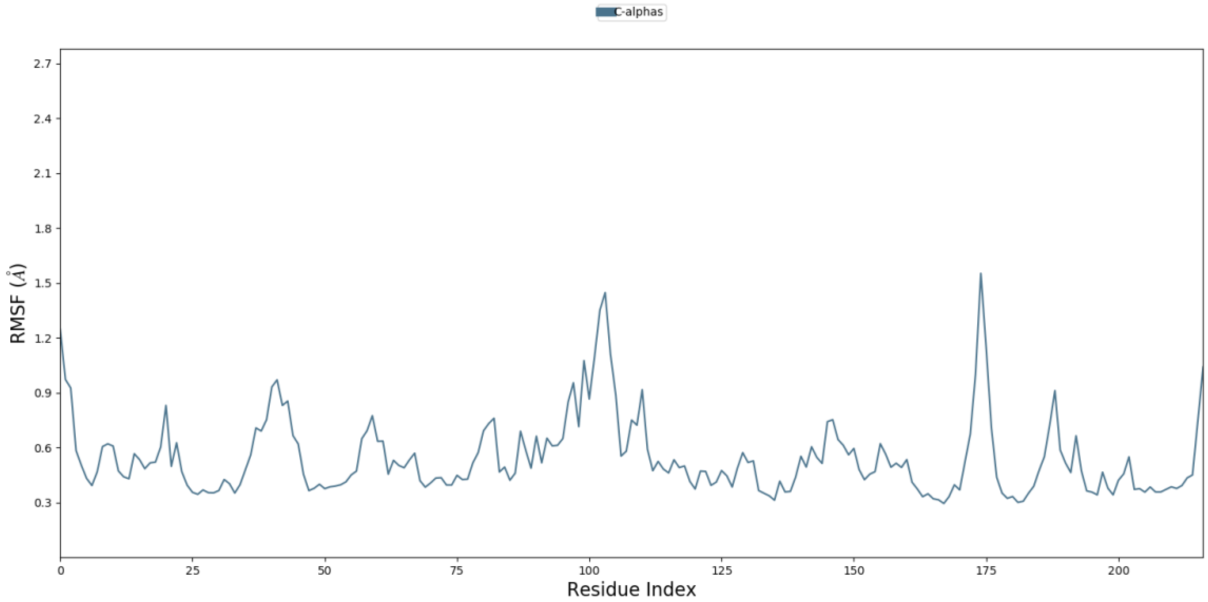

Figure S6: Cathepsin S-iDTBN covalent dock RMSF graph

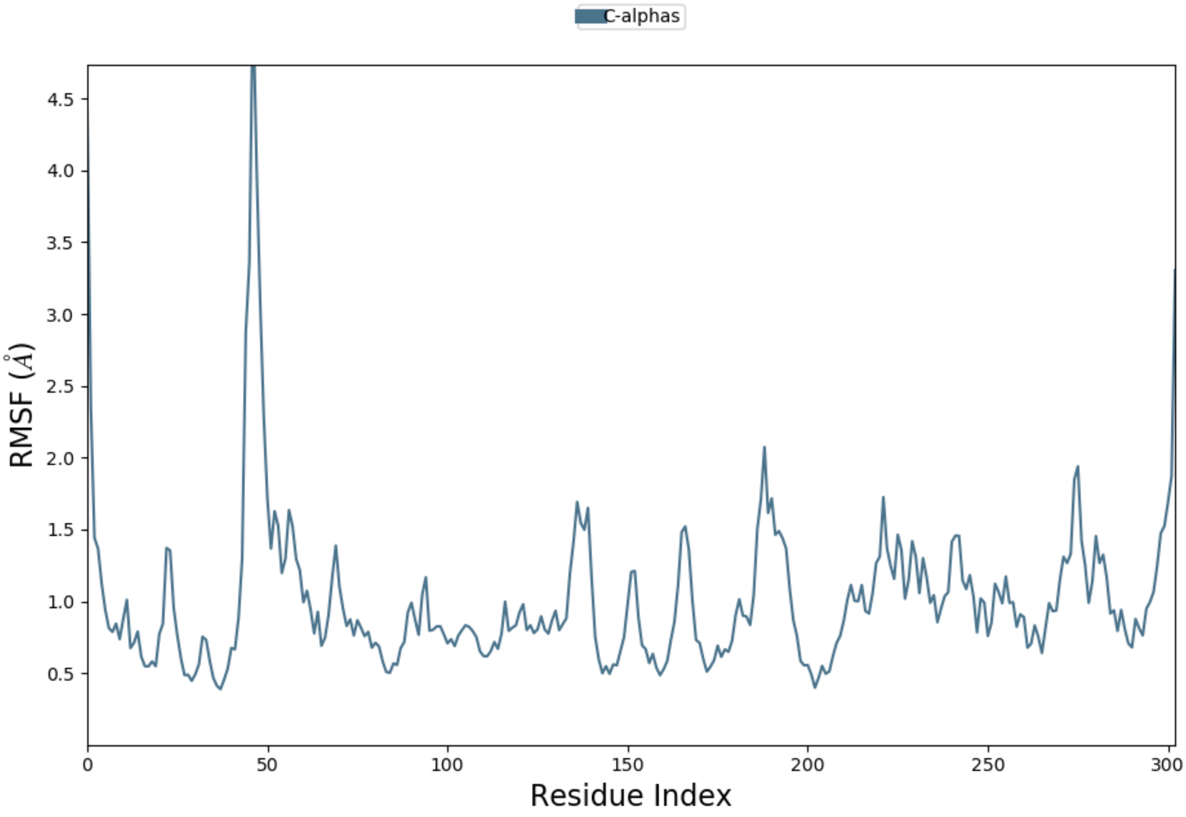

Figure S7: M^{pro}-iDTBN covalent dock RMSF graph

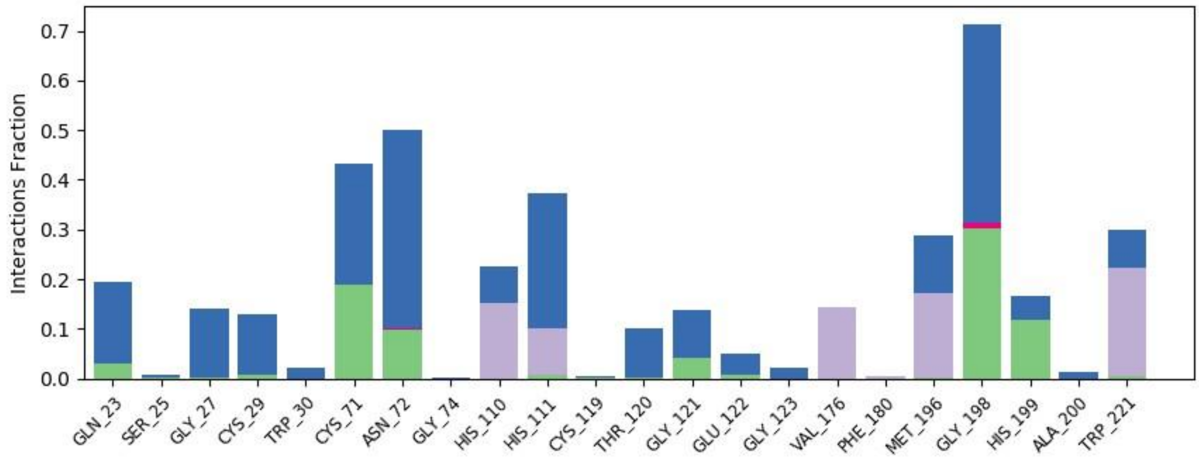

Figure S8: Represents the types of interactions in Cathepsin B–DTBN complex. Blue - water bridge; Green – H-bond; Pink – ionic interaction; Gray – hydrophobic interaction.

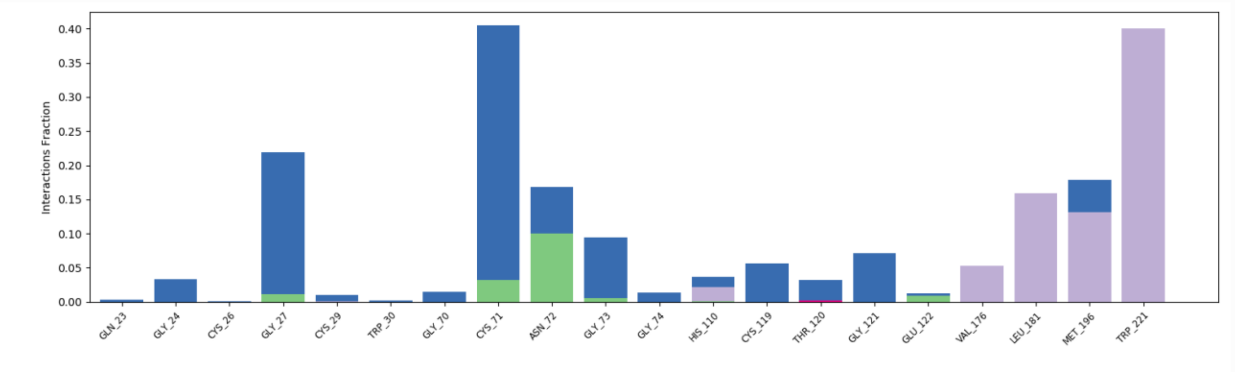

Figure S9: Represent the types of interactions in Cathepsin B – iDTBN complex. Blue - water bridge; Green – H-bond; Pink – ionic interaction; Gray – hydrophobic interaction.

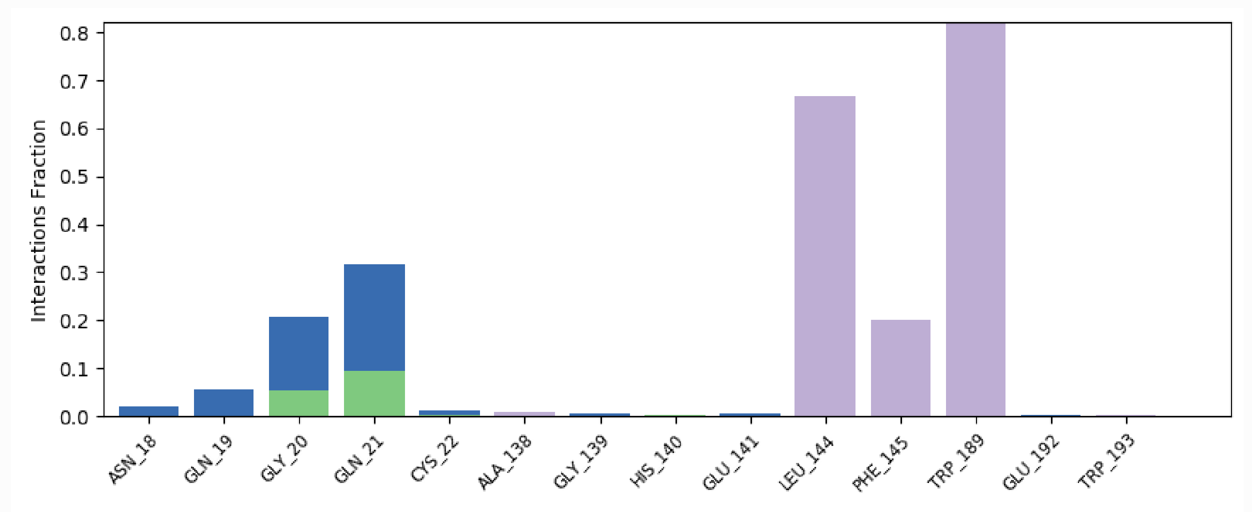

Figure S10: Represent the types of interactions in Cathepsin L – DTBN complex. Blue - water bridge; Green – H-bond; Pink – ionic interaction; Gray – hydrophobic interaction.

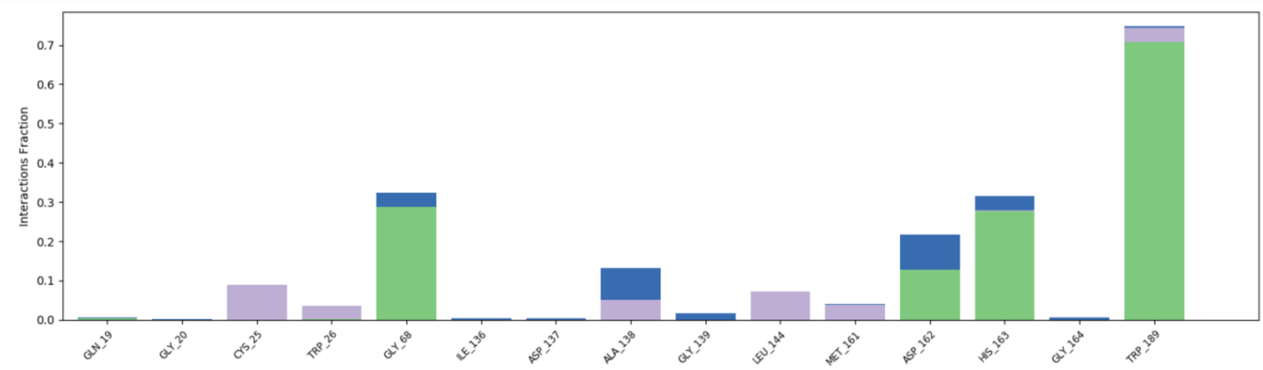

Figure S11: Represent the types of interactions in Cathepsin L – iDTBN complex. Blue - water bridge; Green – H-bond; Pink – ionic interaction; Gray – hydrophobic interaction.

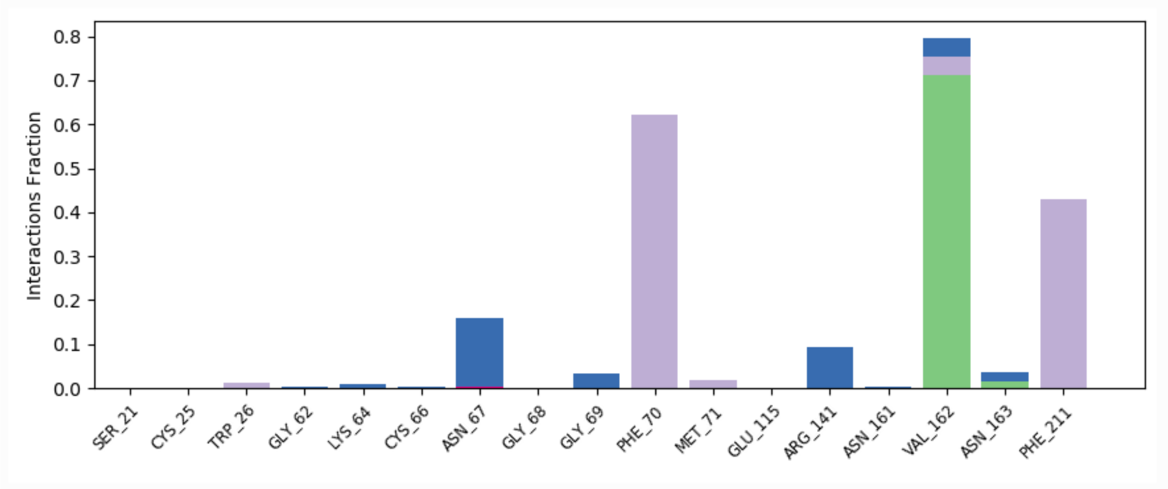

Figure S12: Represent the types of interactions in Cathepsin S – DTBN complex. Blue - water bridge; Green – H-bond; Pink – ionic interaction; Gray – hydrophobic interaction.

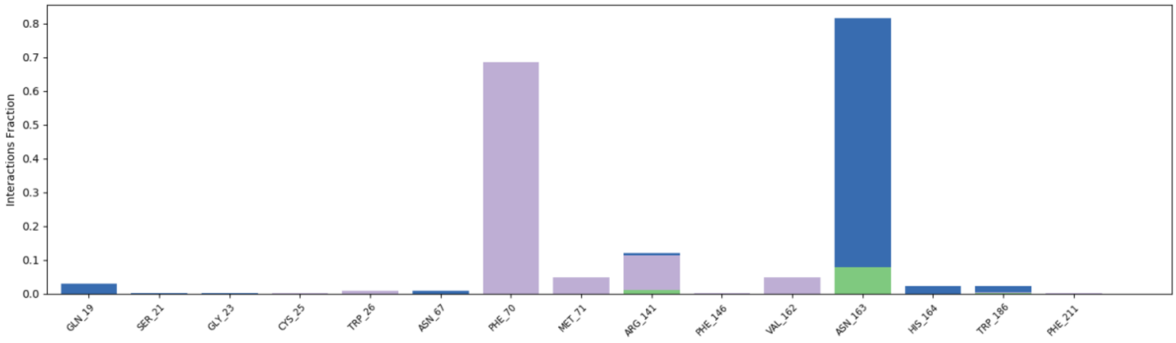

Figure S13: Represent the types of interactions in Cathepsin S–iDTBN complex. Blue - water bridge; Green – H-bond; Pink – ionic interaction; Gray – hydrophobic interaction.

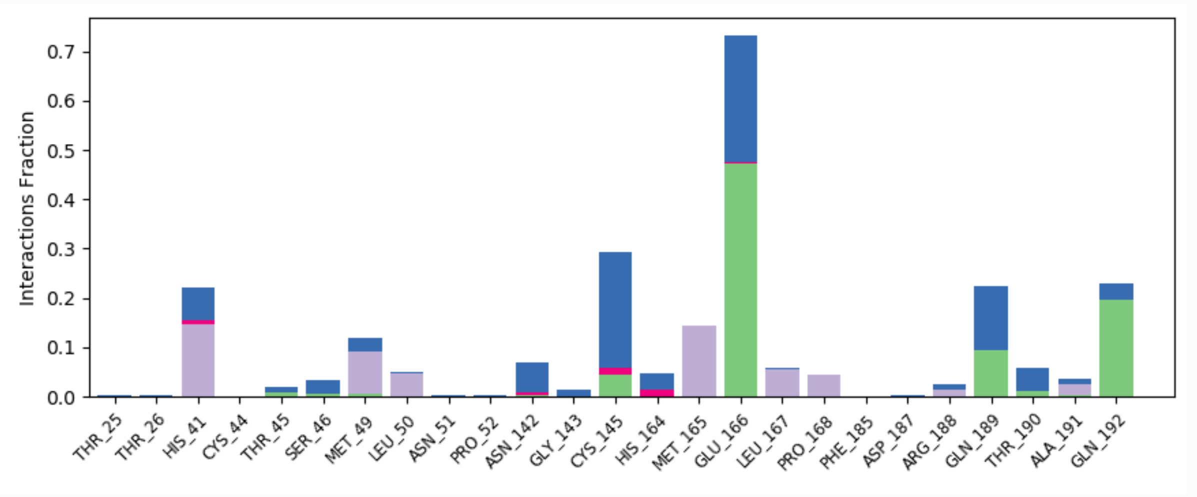

Figure S14: Represent the types of interactions in M^{pro}–iDTBN complex. Blue - water bridge; Green – H-bond; Pink – ionic interaction; Gray – hydrophobic interaction.

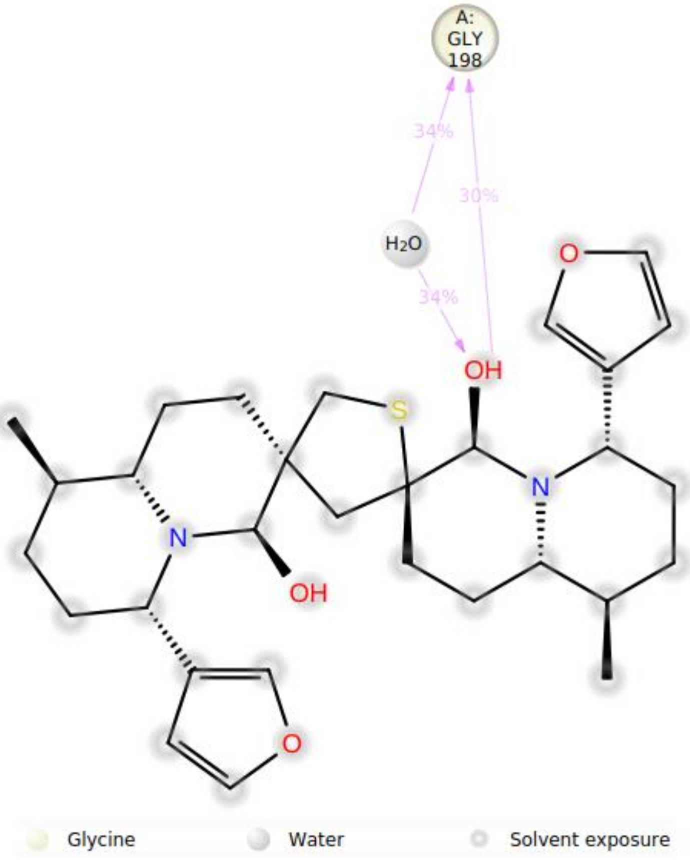

a

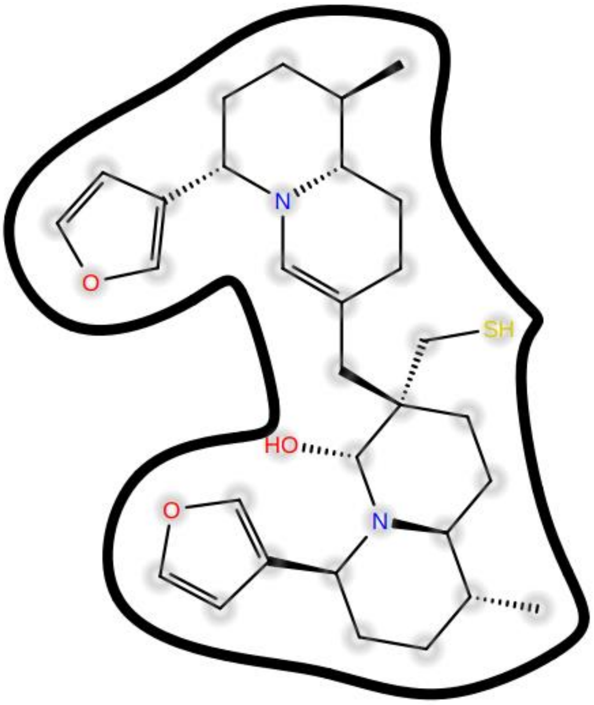

b

Figure S15: 2-D representation of various interactions between a) Cathepsin B and DTBN; b) Cathepsin B and iDTBN; which were persistent ≥ 30% minimum contact strength during 100 ns MD simulation.

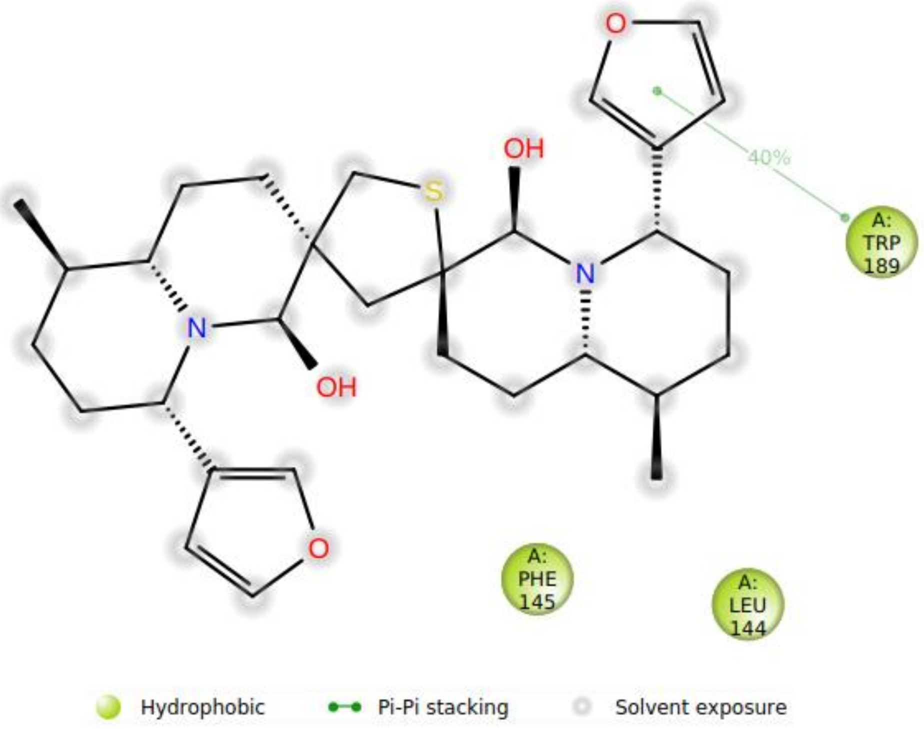

a

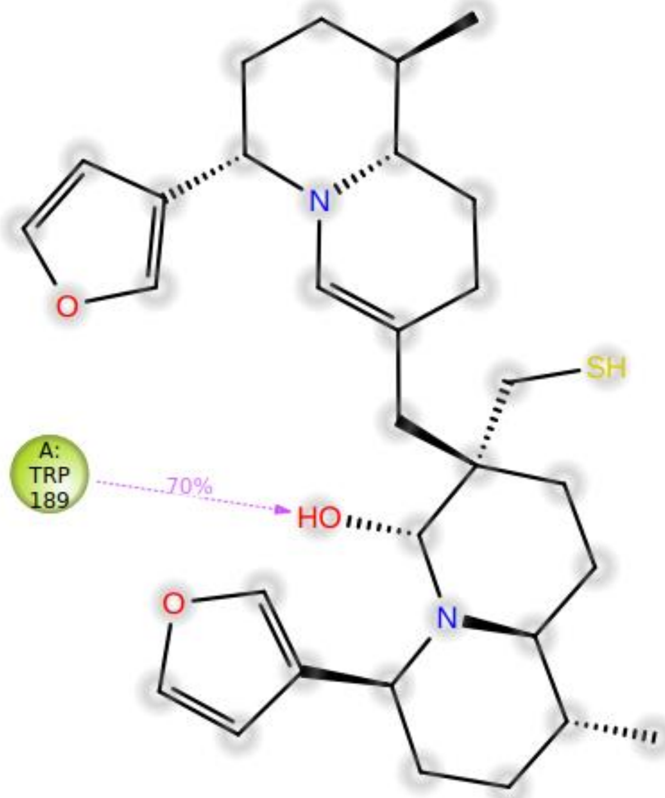

b

Figure S16: 2-D representation of various interactions between a) Cathepsin L and DTBN; b) Cathepsin L and iDTBN; which were persistent ≥ 30% minimum contact strength during 100 ns MD simulation.

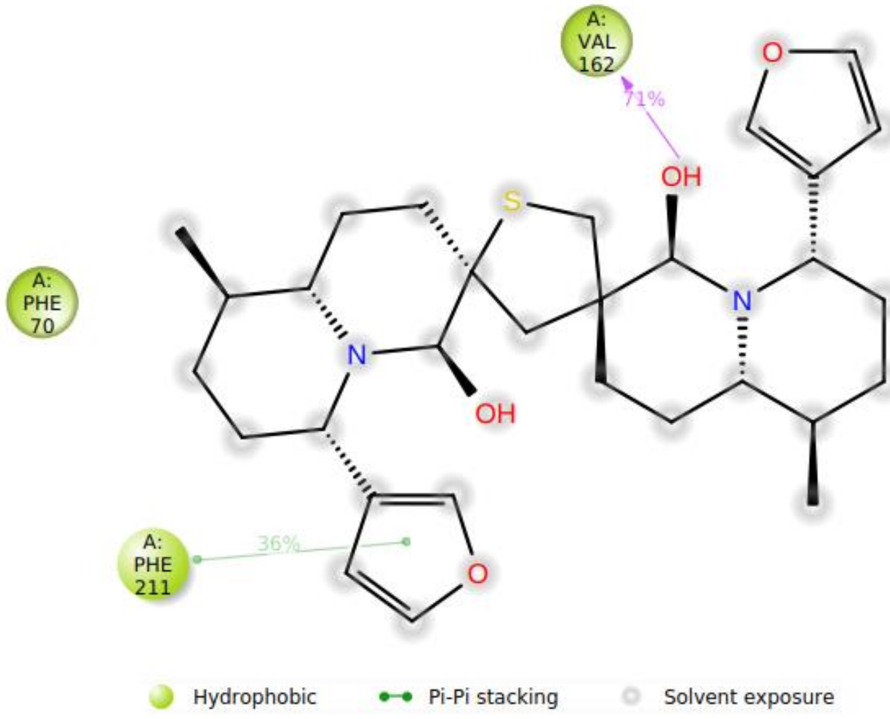

a

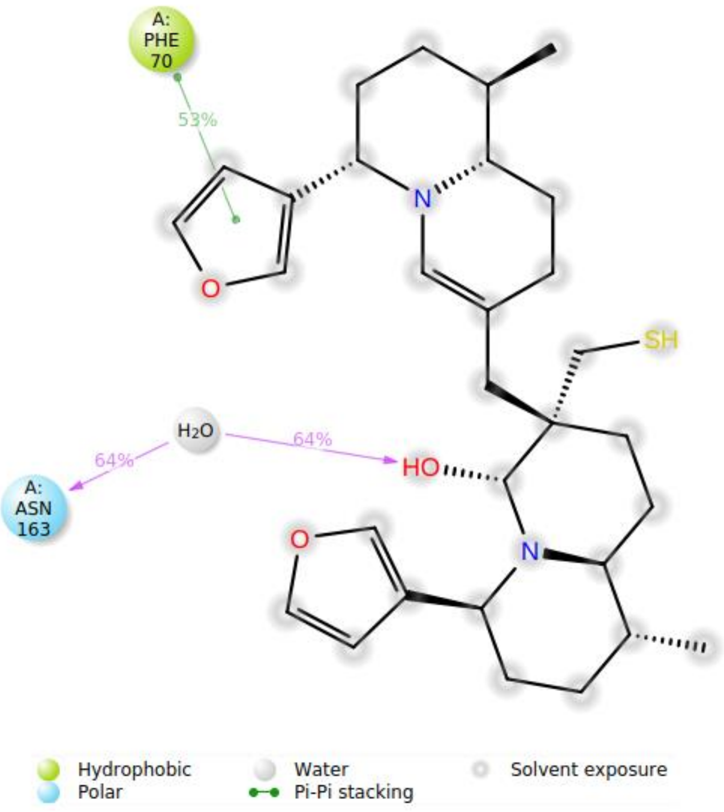

b

Figure 17: 2-D representation of various interactions between a) Cathepsin S and DTBN; b) Cathepsin S and iDTBN; which were persistent ≥ 30% minimum contact strength during 100 ns MD simulation.

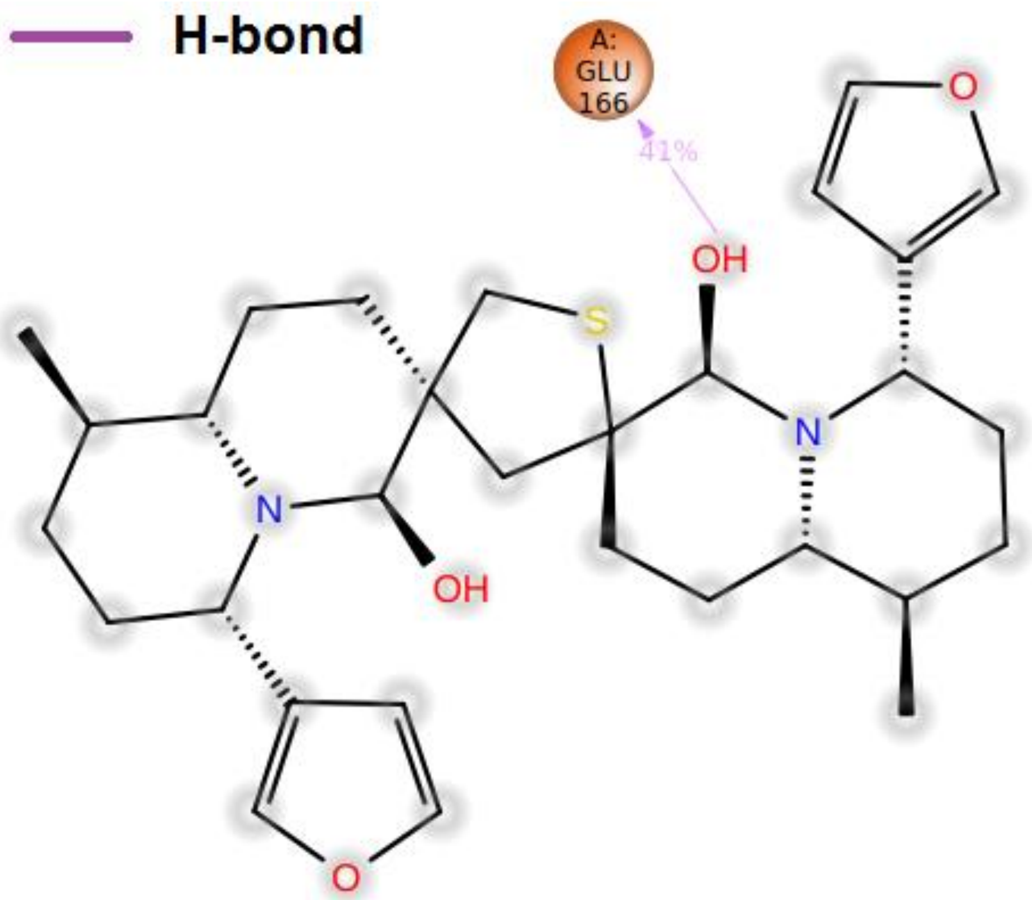

Figure S18: 2-D representation of interactions between a) M[pro] and DTBN which is persistent ≥ 30% minimum contact strength during 100 ns MD simulation.
